# Fluoride Anion Recognition by a Multifunctional Urea Derivative: An Experimental and Theoretical Study

**DOI:** 10.3390/s16050658

**Published:** 2016-05-09

**Authors:** Jana Schiller, Raúl Pérez-Ruiz, Diego Sampedro, Eugenia Marqués-López, Raquel P. Herrera, David Díaz Díaz

**Affiliations:** 1 Institut für Organische Chemie, Universität Regensburg, Universitätsstr. 31, Regensburg 93053, Germany; Jana.Schiller@chemie.uni-regensburg.de (J.S.); raul.perez-ruiz@ur.de (R.P.-R.); 2 Departamento de Química, Universidad de La Rioja, Madre de Dios, 51, Logroño 26006, Spain; diego.sampedro@unirioja.es; 3 Laboratorio de Organocatálisis Asimétrica, Departamento de Química Orgánica, Instituto de Síntesis Química y Catálisis Homogénea (ISQCH), CSIC-Universidad de Zaragoza, Pedro Cerbuna 12, Zaragoza 50009, Spain; mmaamarq@unizar.es (E.M.-L.); raquelph@unizar.es (R.P.H.); 4 Instituto de Química Avanzada de Cataluña-Consejo Superior de Investigaciones Científicas (IQAC-CSIC), Jordi Girona 18-26, Barcelona 08034, Spain

**Keywords:** fluoride, anion recognition, sensor, *N*,*N*′-disubstituted urea, charge-transfer complex, association constant, absorption spectroscopy, fluorescence spectroscopy

## Abstract

In this work we demonstrate the ability of a multifaceted *N*,*N*′-disubstituted urea to selectively recognize fluoride anion (F^−^) among other halides. This additional function is now added to its already reported organocatalytic and organogelator properties. The signaling mechanism relies on the formation of a charge-transfer (CT) complex between the urea-based sensor and F¯ in the ground state with a high association constant as demonstrated by absorption and fluorescence spectroscopy. The nature of the hydrogen bonding interaction between the sensor and F¯ was established by ^1^H-NMR studies and theoretical calculations. Moreover, the recovery of the sensor was achieved by addition of methanol.

## 1. Introduction

Anion sensing and, as consequence, the design, synthesis and development of new sensors constitute nowadays a highly active research field [1,2,3,4,5]. It is well established that anion-sensor coordination takes place usually by hydrogen bonding and/or electrostatic interactions. Among others, sensors with an optical response (*i.e.*, fluorescence) have been found to be the most suitable and attractive tools for anion recognition mainly due to their high sensitivity at low analyte concentration [6]. Within this context, different signaling mechanisms such as photoinduced electron transfer (PET) [7,8,9,10,11], excimer/exciplex formation [12,13], intramolecular charge transfer (ICT) [14,15], and excited-state proton transfer [16,17] have been previously reported in the literature.

After Wilcox [18] and Hamilton [19] discovered the interaction between urea-derivatives and phosphonates, sulphates or carboxylates forming stable 1:1 complexes, the urea moiety has played an essential role as one of the most suitable binding sites in the vast field of anion receptor chemistry. In general, recognition and detection of other anions such as fluoride (F¯) have attracted considerable interest because of its established role in dental care [20], treatment of osteoporosis [21] and its association with chemical weapons (e.g., nerve gases such as sarin, soman and GF chemical warfare agents) or terrorism (e.g., sarin gas was released in the Tokyo subway attack killing 12 people and injuring 5500 in May, 1995) [22,23]. Examples of fluorescent sensors with urea-containing receptor that detect selectively fluoride anion have been reported [24,25,26,27,28,29,30,31,32,33] and explored by computational techniques [34,35,36,37,38,39,40,41].

**Figure 1:**
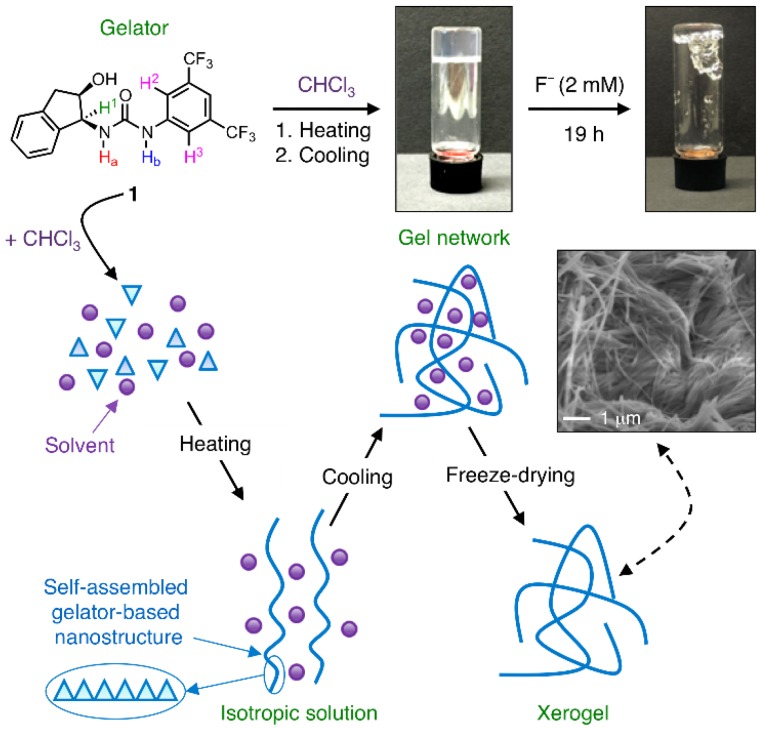
Illustration of the formation of the supramolecular organogel made of urea **1** in chloroform (*c* = 8 g·L^−1^) and its observed disruption upon addition of fluoride. The fibrillar nature of the gel was observed by scanning electron microscopy of the corresponding xerogel.

Recently, we have reported the self-assembly properties of a well-known chiral *N*,*N*′-disubstituted urea-based organocatalyst [42,43] **1** that lead to the formation of multiresponsive, hierarchical supramolecular organogels at low concentrations driven mainly by hydrogen bonding and *π*-*π* interactions (Figure 1) [44]. In general, multifunctional molecular structures have received great attention during the last decade due to their potential use in advanced materials and devices [45]. Herein, we describe a new facet of this versatile urea **1** as a selective fluoride anion receptor among other halides.

## 2. Materials and Methods

### 2.1. Materials

All commercially available solvents and reagents for synthesis and analysis (p.a. grade) were used as received. Urea derivatives were synthesized and characterized as previously described and showed identical spectroscopic data to those reported [44]. Briefly, urea **1** was easily synthesized by an equimolar reaction of commercial (1*S*,2*R*)-1-amino-2,3-dihydro-1*H*-inden-2-ol and 3,5-bis(trifluoromethyl)phenyl isocyanate in methylene chloride at room temperature [44]. The organogel was prepared as following: urea **1** (8 mg, 0.02 mmol) was weighed and placed into a screw cap vial (4.5 cm length × 1.2 cm diameter) and 1 mL of CHCl_3_ was added. The closed vial was gently heated with a heat gun until the solid compound was completely dissolved. The obtained isotropic solution was cooled down spontaneously to room temperature affording the gel formation. No flow of the material was observed upon turning the vial upside-down at room temperature.

### 2.2. Methods

#### 2.2.1. Absorption and Fluorescence Spectroscopy

Absorption spectra were recorded using a Cary 50 Bio UV-visible spectrophotometer (Varian, Palo Alto, CA, USA). Fluorescence and excitation spectra were carried out using a Fluoromax-4 spectrofluorometer (Horiba, Kyoto, Japan). The excitation and emission slit widths were 5 nm. The samples were placed into quartz cells of 1 cm path length. All measurements were performed at room temperature and compound concentrations were fixed as indicated.

#### 2.2.2. Theoretical Calculations

Chemical structures were optimized with the GAUSSIAN09 program package [46] and the density functional theory (DFT) method. B3LYP functional [47] together with the standard basis set 6–31G(d) [48] and the CPCM-SCRF method [49,50] were used to model the solvent (acetonitrile). NMR shifts were computed on the optimized structures using the GIAO method [51].

#### 2.2.3. NMR Spectroscopy

NMR spectra were recorded at 400 MHz on an AVANCE-II instrument (Bruker, Billerica, MA, USA).

#### 2.2.4. Electron Microscopy

Electron microscopy images were obtained with a Merlin field emission scanning electron microscope (FESEM, resolution = 0.8 mm resolution, Carl Zeiss, Jena, Germany) equipped with a digital camera and operating at 10 kV (accelerating voltage) and 10 mA (emission current). The sample was prepared by freeze-drying the corresponding organogel. Prior to imaging, a 5 nm sized Pt film was sputtered (40 mA, 30 s) on the sample placed on carbon tape.

## 3. Results and Discussion

As mentioned above, urea-based compound **1** can not only function as a good organocatalyst for Friedel-Crafts alkylations [42,43], but also as a molecular building block for the bottom-up preparation of multiresponsive supramolecular gels in several organic solvents at concentrations ranging from 3 to 50 g·L^−1^ (Figure 1) [44]. During our preliminary studies we observed that a gel made from **1** in chloroform (*c* = 8 g·L^−1^) collapsed in the presence of fluoride anions (Figure 1). Similar behavior has been already reported with other gels, in which the addition of halide anions causes the disruption of intermolecular hydrogen bonding leading to either *gel*-to-*sol* transitions and/or colorimetric *gel*-to-*gel* transitions [52,53,54,55,56,57,58]. Furthermore, changes in the absorption spectra of **1** in the presence of halides in chloroform solution clearly appeared (Figure S1), confirming the host-guest binding in all cases. At this point, we decided to focus our attention on the complete photophysical characterization of the potential molecular interaction between **1** and halide anions, as well as on the anion sensing signaling mechanism and selectivity of the process.

The absorption spectrum of sensor **1** in the absence of halide anions was first measured in different solvents and showed two bands that corresponded to the two characteristic electronic transitions, ππ* at higher energy and nπ* transition at lower energy. Table 1 summarizes the photophysical data obtained for compound **1** in different solvents (for a comparison with various urea analogues, see Figures S2–S9 and Tables S1–S3). A red-shifted emission of 21 nm as well as an increase in the Stokes shift values when increasing solvent polarity (DMSO *vs.* CHCl_3_) was observed. On the other hand, a protic solvent provided practically the same results than a polar aprotic solvent (MeOH *vs.* MeCN, respectively). These results suggested an enhancement of the overall dipole moment on excitation albeit almost independent of the solvent.

Having established the photophysical data of compound **1** in different solvents, we tested its ability to recognize halide anions in a suitable solvent. In this sense, DMSO possesses an absorption band in the range of 200–280 nm, which would interfere with the excitation in the fluorescence experiments (250 nm). MeOH was discarded because it is usually used for checking the reversibility of the recognition process. Thus, we decided to do the next studies in MeCN because it is a relatively inert solvent without any absorption band in the region between 210 and 600 nm and it is commonly used in photochemical/photophysical investigations. On the other hand, we used tetrabutylammonium (TBA) halides for our studies because they have good solubility in MeCN and TBA is one of the most common counterions used in this field [59,60,61]. Other counterions such as K^+^ and Na^+^ were not suitable for these studies because they can also be coordinated with the urea moiety [61].

**Figure 2:**
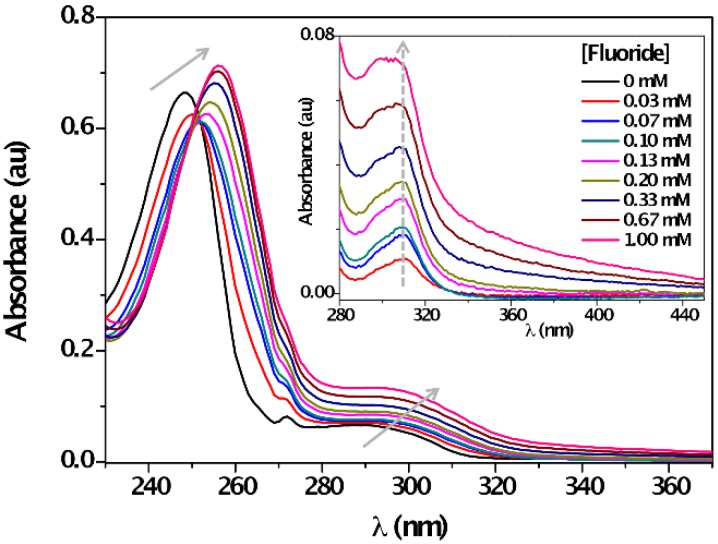
Absorption spectra of **1** (*c* = 0.04 mM) in the absence and with increasing amounts of F¯ (*c* = 0, 0.07 mM→1 mM) in acetonitrile at room temperature. Inset: Difference UV-spectra of [**1** + F¯] – **1** in the long wavelength region.

The UV-vis absorption spectra of **1** in MeCN in the absence of F¯ showed two bands centered at 249 nm and 290 nm. Upon titration with F¯, the ground state was affected; a bathochromic shift due to anion recognition on the two absorption maxima together with the appearance of an isosbestic point at 252 nm, clearly pointed out to the formation of new species (Figure 2). The reverse was true for other halide anions (*i.e.*, Cl¯, Br¯, I¯) as slight-to-negligible changes in the UV-vis spectra in MeCN were observed upon addition of these species (Figure S10).

The absorbance intensity of the ππ* transition decreased, whereas nπ* transition band remarkably enhanced. These changes confirmed the formation of a new species, named charge-transfer (CT) complex with new photophysical properties after the anion recognition. To examine the formation of the CT complex, difference spectra (*i.e.*, Abs_[**1**+F¯]_–Abs**_1_**) were obtained. The results showed the emerging of a new band at *ca.* 310 nm (Figure 2, inset), which was attributed to the CT complex absorption maximum. The formation constant of a CT complex (K_CT_) was estimated spectro-photometrically by the Benesi–Hildebrand procedure (Equation (1)) [62]:

[1]/Abs_CT_ = [1/(K_CT_ ε_CT_ [F¯])] + (1/ε_CT_) (1)

**Figure 3:**
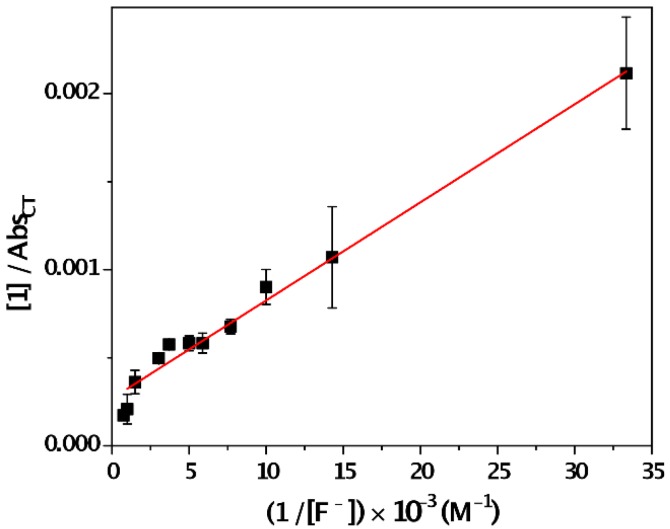
Benesi-Hildebrand plot used to determine the association constant of the CT complex formed by sensor **1** and F¯ (λ_max_ = 300 nm) at different fluoride concentrations ([**1**] = 0.02 mM).

The corresponding absorbance/concentration plot is shown in Figure 3. From Equation (1), Abs_CT_ and ε_CT_ represent the absorbance due to the CT band at 300 nm at different concentrations of F¯ and the molar absorption coefficient of the CT complex, respectively. A value of 3721 M^−1^·cm^−1^ ± 340 M^−1^·cm^−1^ for the ε_CT_ was obtained from the intercept in acetonitrile (log ε_CT_ = 3.6). Consequently, the corresponding K_CT_ (slope) was found to be 4826 M^−1^ ± 111 M^−1^. This moderate-high value of K_CT_ indicates a strong intermolecular interaction between **1** and F¯ in the ground state. This behavior was not observed in the presence of other halides such as Cl¯, Br¯ and I¯ (*vide infra*).

**Figure 4:**
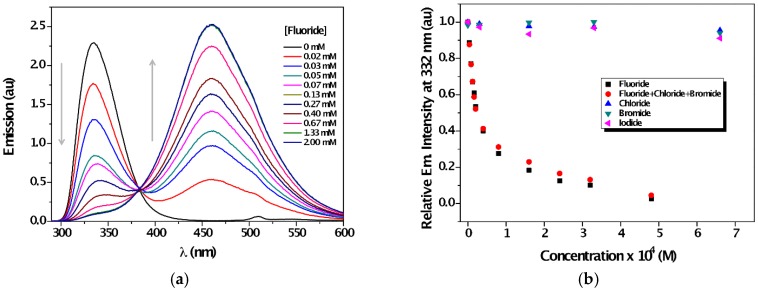
(**a**) Emission spectra of **1** (*c* = 0.04 mM, λ_exc_ = 252 nm) in the presence of increasing amounts of F¯ (*c* = 0, 0.02 mM→2 mM) in acetonitrile; (**b**) Changes in the emission at 332 nm upon titration with different halides.

In order to observe changes in the excited state, the fluorescence of **1** in the presence of increasing amounts of anions was investigated. In agreement with the marked variations detected in the ground state, emission of **1** was fully quenched by the presence of F¯ (Figure 4a), whereas a new comprehensive band with a maximum at *ca.* 460 nm appeared with the formation of an isoemissive point at 383 nm. On the other hand, the maximum emission intensity of **1** showed no changes in the presence of Cl¯, Br¯ and I¯ (Figure 4b, Figure S11 and Figure S12), which demonstrated the preference of sensor **1** for F¯. Furthermore, the detection limit of fluoride anion was established at around 0.003 mg·L^−1^ (see ESI, Figure S13); this fluoride concentration is categorized as low-fluoride water (up to 0.5 mg·L^−1^) [63]. Other related ureas, albeit lacking gelation ability, were found to be also suitable for sensing selectively fluoride anion among other halides (Figure S14). These results suggest that, at least in this case, the self-assembly tendency of the sensor does neither hinder nor favor its anion sensing properties. In addition, sensor **1** showed high affinity for other anions such acetate and phosphate, while the reverse was true for hydrosulfate (Figure S15).

A static fluorescence quenching of sensor **1** seemed to occur since the Stern–Volmer plot presented a non-linear behavior at high amounts of fluoride (Figure S16). For comparison, a strong non-nucleophilic base such as 1,8-diazabicyclo[5.4.0]undec-7-ene (DBU) was also used. In this case, fluorescence quenching followed a clearly fitted-linearity, in contrast than the one found for fluoride (Figure S16). In addition, ^1^H-NMR experiments showed that urea protons completely disappeared in the presence of DBU (Figure S17). Therefore, the different effects obtained for the emission and ^1^H-NMR of **1** in the presence of DBU and fluoride, support the formation of a CT complex between sensor **1** and fluoride.

**Figure 5:**
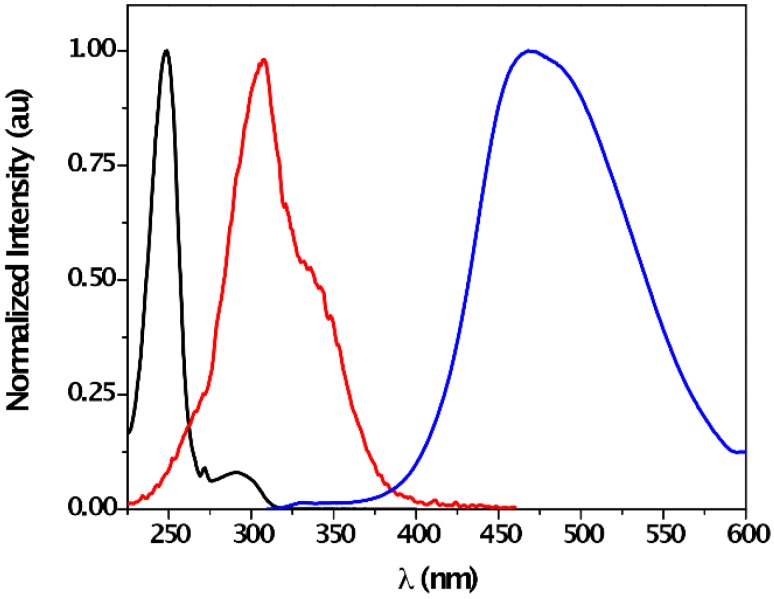
Normalized absorption band of **1** (black line, *left*), excitation (red line, *centered*, λ_em_ = 468 nm) and emission (blue line, *right*, λ_exc_ = 320 nm) spectra of a mixture of **1** (*c* = 0.04 mM) and F¯ (*c* = 2 mM) in acetonitrile under aerobic conditions.

In order to establish the formation of [**1**---F]¯ complex, steady-state fluorescence measurements were performed with a solution of **1** and F¯ in acetonitrile (Figure 5). Upon selective CT complex excitation at 320 nm (sensor **1** does not absorb in this region), its emission was detected, with a maximum at *ca.* 468 nm. In good agreement with the UV-absorption measurement, the corresponding excitation spectrum showed a maximum at 308 nm.

**Figure 6:**
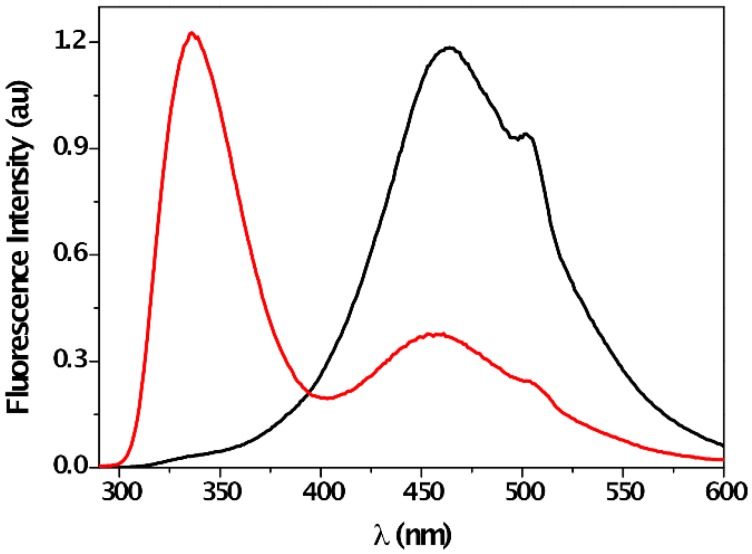
Emission spectra (λ_exc_ = 320 nm) of a mixture of **1** (*c* = 0.04 mM) and F¯ (*c* = 2 mM) in acetonitrile in the absence (black line, *right*) and in the presence of 10% methanol (red line, *left*).

The [**1**---F]¯ complex formation through H-bonding interaction between F¯ and the urea moiety was further confirmed by ^1^H-NMR titration experiments in CD_3_CN. The urea protons H_a_ and H_b_ appeared at 5.94 ppm and 7.92 ppm (Figure S18). In the presence of increasing equivalents of F¯, the urea resonances were gradually shifted to downfield by *ca.* 2 ppm and *ca.* 4 ppm, respectively, reflecting H-bond formation between receptor and anion. In addition, some effects on aromatic substituents (for instance, polarization-induced of the C-H bonds via a through-space effect) obtaining downfield shift due to deshielding effect by partial positive charge formed onto the proton [32], could be induced by this H-bonding interaction. In fact, this electrostatic effect was also detected in aromatic protons H^2^ and H^3^ showing a weak downfield shift upon addition of F¯ equivalents (Figure S18), in good correlation with literature data [30,60]. Moreover, deprotonation was not involved in the signaling mechanism, as reflected by the incomplete disappearance of the H_a_ signal, even at high anion concentration. This suggests that the N-H amide bonding length increased during the anion recognition, which was confirmed by the multiplicity of H^1^, which changed from a double doublet in the absence of F¯ to a doublet upon addition of one equivalent of F¯ (Figure S18). Furthermore, the effect of protic solvent on the fluorescence of [**1**---F]¯ complex also confirmed the H-bonding nature of this interaction. The addition of methanol to a mixture of **1** and F¯ in acetonitrile led to the recovery of the emission band (Figure 6), which clearly supported the reversibility of the process by the interaction between the urea moiety and the protic solvent (see Figure S17 for additional ^1^H-NMR experiments).

**Figure 7:**
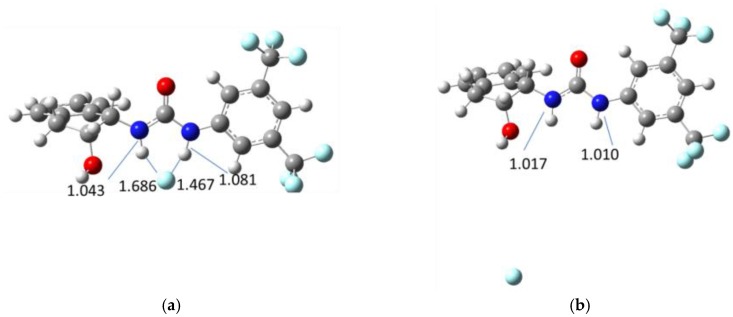
Geometries of sensor **1** fluoride bonded (**a**) and non-bonded fluoride (**b**).

The foregoing experimental results pointed out that the signaling mechanism involved a CT complex formation through H-bonding interaction between **1** and F¯. To further support this conclusion, computational calculations at the B3LYP/6-31G(d) level of theory using the CPCM method (acetonitrile as solvent) were carried out. Optimization of the geometries in the absence and presence of F¯ was performed (Figure 7) and the corresponding N-H bond distances were calculated. The values for N-H_a_ and N-H_b_ bond lengths without anion were found to be 1.017 Å and 1.010 Å, respectively. After fluoride binding complex optimization, the values of these bond distances were 1.043 Å for N-H_a_ bond and 1.081 Å for N-H_b_ bond. Although, bond-elongation was observed in both cases, it seemed not to be sufficient for hydrogen abstraction by F¯. The H---F distances were 1.686 Å (H_a_---F) and 1.467 Å (H_b_---F), which were close to the experimental values reported in the literature [60,64] for complexation of analogous compounds with F¯ ions. Similar results were obtained for the analogous ureas (Figures S19–S21).

Moreover, both N-H_a_ and N-H_b_ GIAO-NMR shifts (δ) were also calculated in the absence/presence of fluoride anion. Upon addition of F¯, a strong shift to downfield was found for both hydrogen atoms, especially for H_b_. The computed signal for H_a_ appeared at 9 ppm, while the computed signal for H_b_ appeared at 13 ppm. These data are in good agreement with those observed experimentally. Thus, the qualitative picture of the molecular recognition is well represented by the computational data. Overall, these computational results are in concordance with experimental observations where the formation of a complex between sensor **1** and F¯ in the ground state prevails over a possible deprotonation of the urea moiety.

## 4. Conclusions

**Figure 8:**
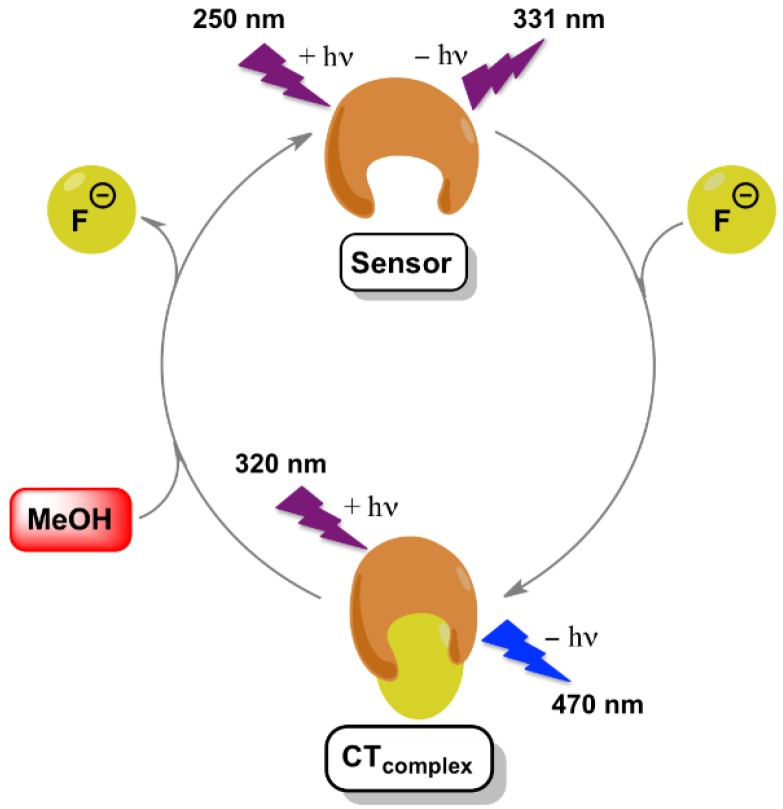
Illustration of the signaling mechanism of F¯ by sensor **1**.

In summary, urea **1** was found to selectively recognize fluoride anion among other halides as demonstrated by means of absorption as well as fluorescence spectroscopic data. The signaling mechanism relies on the formation of a CT complex between sensor **1** and F¯ in the ground state (Figure 8). In this sense, upon recognition of the anion, new absorption and fluorescence bands of this CT complex at longer wavelengths were clearly detected together with a high association constant. The nature of the H-bonding interaction between **1** and fluoride was unambiguously proven by ^1^H-NMR studies and theoretical calculations. Finally, the recovery of the sensor was achieved using protic solvents such as methanol.

## Figures and Tables

**Table 1:** Photophysical data of sensor **1**. ^1^

Solvent	λ_abs_ (nm) (ππ*/nπ*)	log ε (M^−1^·cm^−1^) (ππ*/nπ*)	λ_em_ (nm)	Stokes (cm^−1^)	Singlet Energy (eV)
CHCl_3_	243/287	4.39/3.34	324	3149	4.07
DMSO	-/297	-/3.50	345	4237	3.87
MeCN	249/291	4.38/2.80	332	3324	3.98
MeOH	249/291	4.42/3.09	335	3640	3.99

^1^ Sensor concentration, [**1**] = 0.01 mM.

## Supplementary Materials

The following are available online at www.mdpi.com/link: Figure S1: Absorption spectra of **1** (0.04 mM) in chloroform in the presence of increasing amounts of the corresponding halide. Figure S2: Absorption spectra of **1** (0.01 mM) in chloroform, acetonitrile, methanol and dimethylsulfoxide at room temperature under aerobic conditions. Figure S3: Normalized excitation and emission spectra of **1** (0.01 mM) in chloroform, acetonitrile, methanol and dimethylsulfoxide at room temperature under aerobic conditions. Figure S4: Absorption spectra of **2** (0.01 mM) in dichloromethane, acetonitrile, methanol and dimethylsulfoxide at room temperature under aerobic conditions. Figure S5: Normalized excitation and emission spectra of **2** (0.01 mM) in dichloromethane, acetonitrile, methanol and dimethylsulfoxide at room temperature under aerobic conditions. Figure S6: Absorption spectra of **3** (0.01 mM) in dichloromethane, acetonitrile, methanol and dimethylsulfoxide at room temperature under aerobic conditions. Figure S7: Normalized excitation and emission spectra of **3** (0.01 mM) in dichloromethane, acetonitrile, methanol and dimethylsulfoxide at room temperature under aerobic conditions. Figure S8: Absorption spectra of **3** (0.01 mM) in dichloromethane, acetonitrile, methanol and dimethylsulfoxide at room temperature under aerobic conditions. Figure S9: Normalized excitation and emission spectra of **3** (0.01 mM) in dichloromethane, acetonitrile, methanol and dimethylsulfoxide at room temperature under aerobic conditions. Table S1: Photophysical data of sensor **2** (*c* = 10^−5^ M). Table S2: Photophysical data of sensor **3** (*c* = 10^−5^ M). Table S3: Photophysical data of sensor **4** (*c* = 10^−5^ M). Figure S10: Absorption spectra of 1 (*c* = 0.04 mM) in the absence and with increasing amounts of Cl¯, Br¯ and I¯ (*c* = 0, 0.03 mM→1 mM) in acetonitrile at room temperature. Figure S11: Emission spectra of **1** (0.04 mM) in the presence of increasing amounts of chloride (λ_exc_ = 252 nm), bromide (λ_exc_ = 252 nm) and iodide (λ_exc_ = 300 nm) in acetonitrile. Figure S12: Emission spectra of **1** (*c* = 0.04 mM, λ_exc_ = 252 nm) in the presence of increasing amounts of a mixture of halide anions (*c* = 0, 0.004 mM→0.48 mM) in acetonitrile. Figure S13: Emission spectra of **1** (*c* = 0.04 mM, λ_exc_ = 252 nm) in the presence of increasing amounts of fluoride (*c* = 0, 0.004 mM → 0.02 mM) in acetonitrile. Figure S14: Changes in the maximum emission of sensors **2**–**4** upon titration with different halides in acetonitrile at room temperature. Figure S15: Changes in the emission of **1** (*c* = 0.04 mM, λ_exc_ = 252 nm) at 332 nm upon titration with different anions in acetonitrile. Figure S16: Stern-Volmer plots for the fluorescence of **1** (*c* = 0.04 mM, λ_exc_ = 252 nm) upon fluoride (■) and DBU (●) titration (*c* = 0, 0.004 mM → 0.32 mM) in acetonitrile. Figure S17: Changes in the ^1^H-NMR (400 MHz) spectra in CD_3_CN. A: sensor **1** (0.02 mmol); B: sensor **1** + 0.75 eq. of fluoride; C: sensor **1** + 0.75 eq. of fluoride + one drop of MeOH; D: sensor **1** + one drop of DBU, Cartesian coordinates for Compounds **1**–**4**. Figure S18: Changes in the ^1^H-NMR (300 MHz) spectra of **1** in CD_3_CN upon addition of F¯. Figures S19–S21: Geometries of sensors **2**–**4** fluoride bonded and non-bonded fluoride.

## Abbreviations

The following abbreviations are used in this manuscript:

CPCM	Conductor-like polarizable continuum model
CT	Charge-transfer
DBU	1,8-Diazabicyclo[5.4.0]undec-7-ene
DMSO	Dimethyl sulfoxide
FESEM	Field emission scanning electron microscopy
GIAO	Gauge-independent atomic orbital
ICT	Intramolecular charge transfer
MeCN	Acetonitrile
MeOH	Methanol
NMR	Nuclear magnetic resonance
PTET	Photoinduced electron transfer
SCRF	Self-consistent reaction field
UV-vis	Ultraviolet-visible
