# Supplementary material for: Fluoride Anion Recognition by a Multifunctional Urea Derivative: An Experimental and Theoretical Study
Source: Sensors (Basel). 2016 May 9;16(5):658. doi: 10.3390/s16050658 (PMC4883349; doi:10.3390/s16050658)
Supplement: Supplementary file 1 [file sensors-16-00658-s001.pdf]

Supplementary Materials: Fluoride Anion Recognition by a Multifunctional Urea: An Experimental and Theoretical Study

Jana Schiller, Raúl Pérez-Ruiz, Diego Sampedro, Eugenia Marqués-López, Raquel P. Herrera and David Díaz Díaz

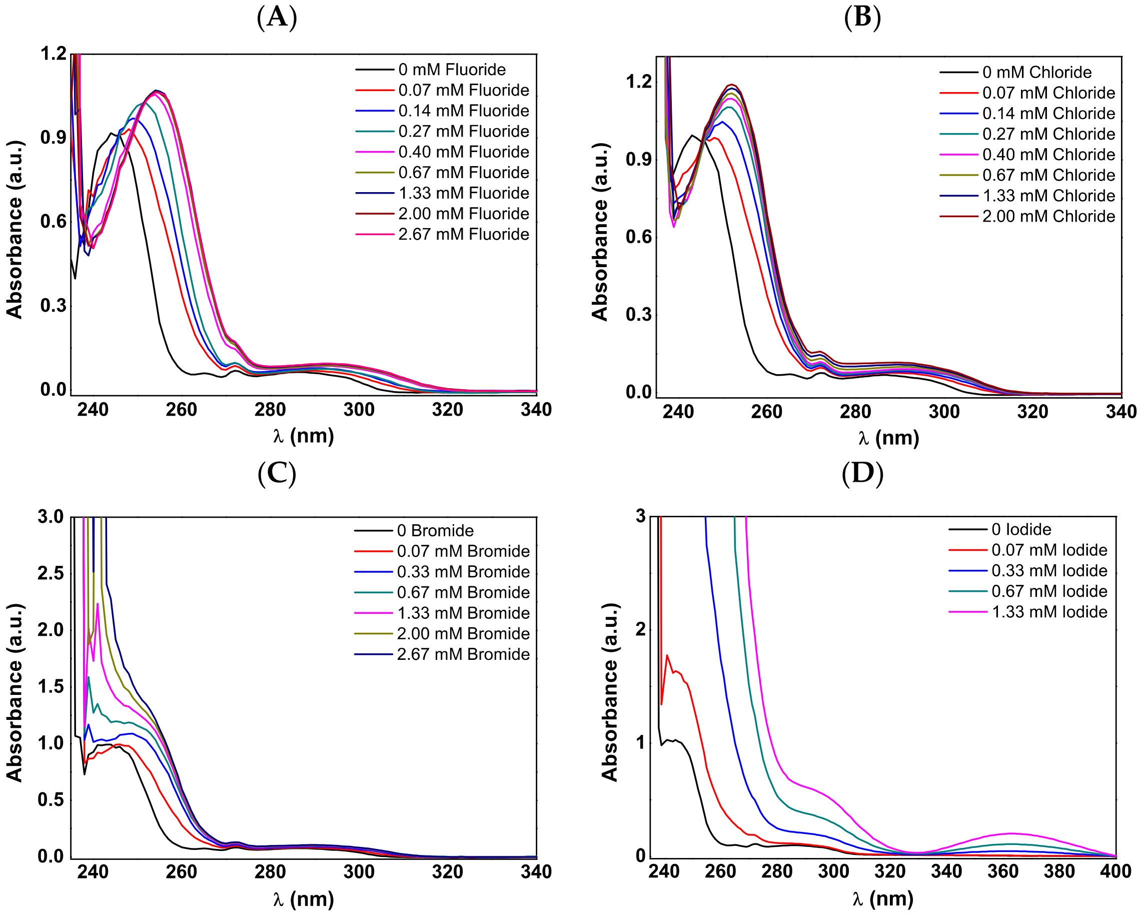

Figure S1. Absorption spectra of **1** (0.04 mM) in chloroform in the presence of increasing amounts of the corresponding halides: (**A**) Fluoride, (**B**) chloride, (**C**) bromide, (**D**) iodide.

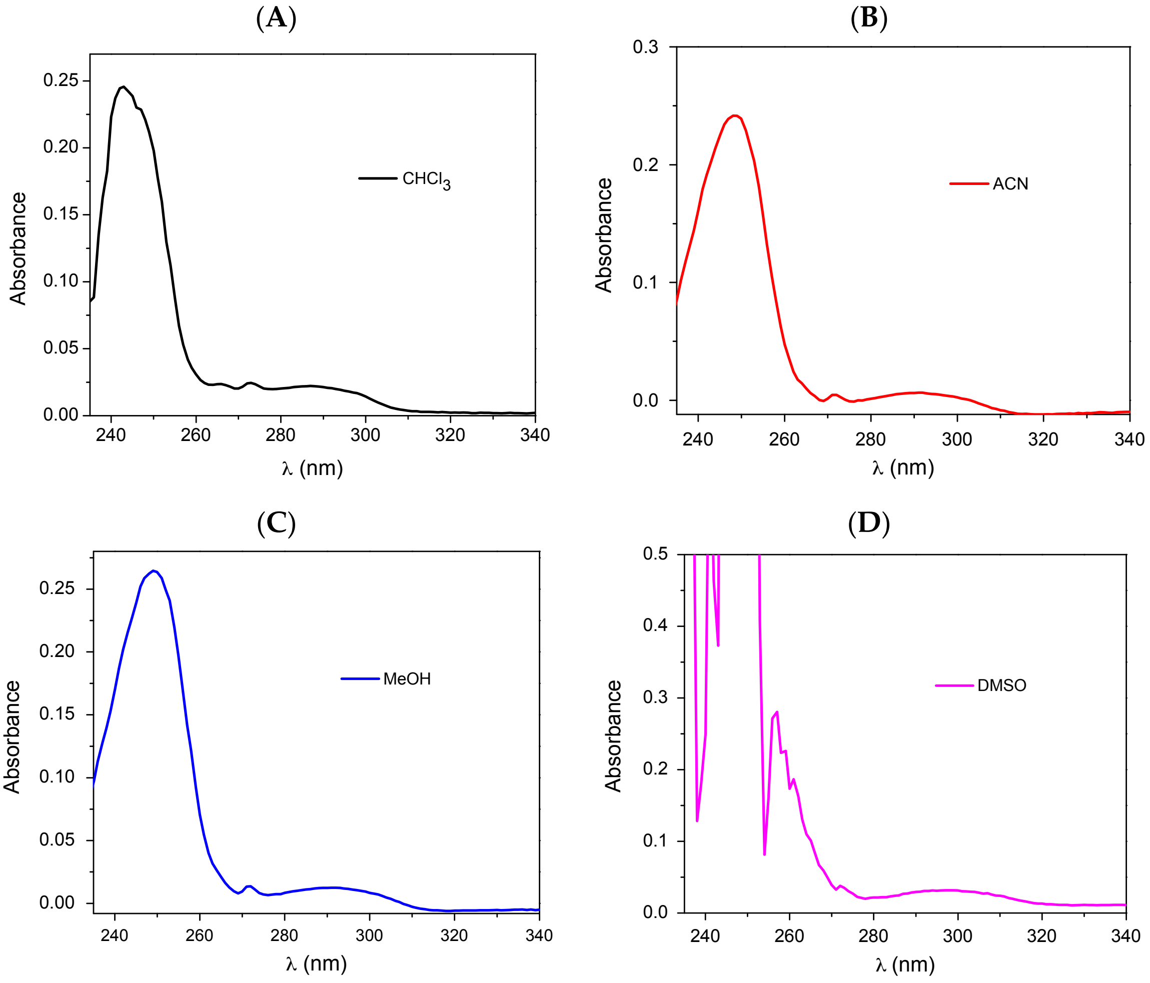

Figure S2. Absorption spectra of **1** (0.01 mM) in (**A**) chloroform, (**B**) acetonitrile, (**C**) methanol and (**D**) dimethylsulfoxide at room temperature under aerobic conditions.

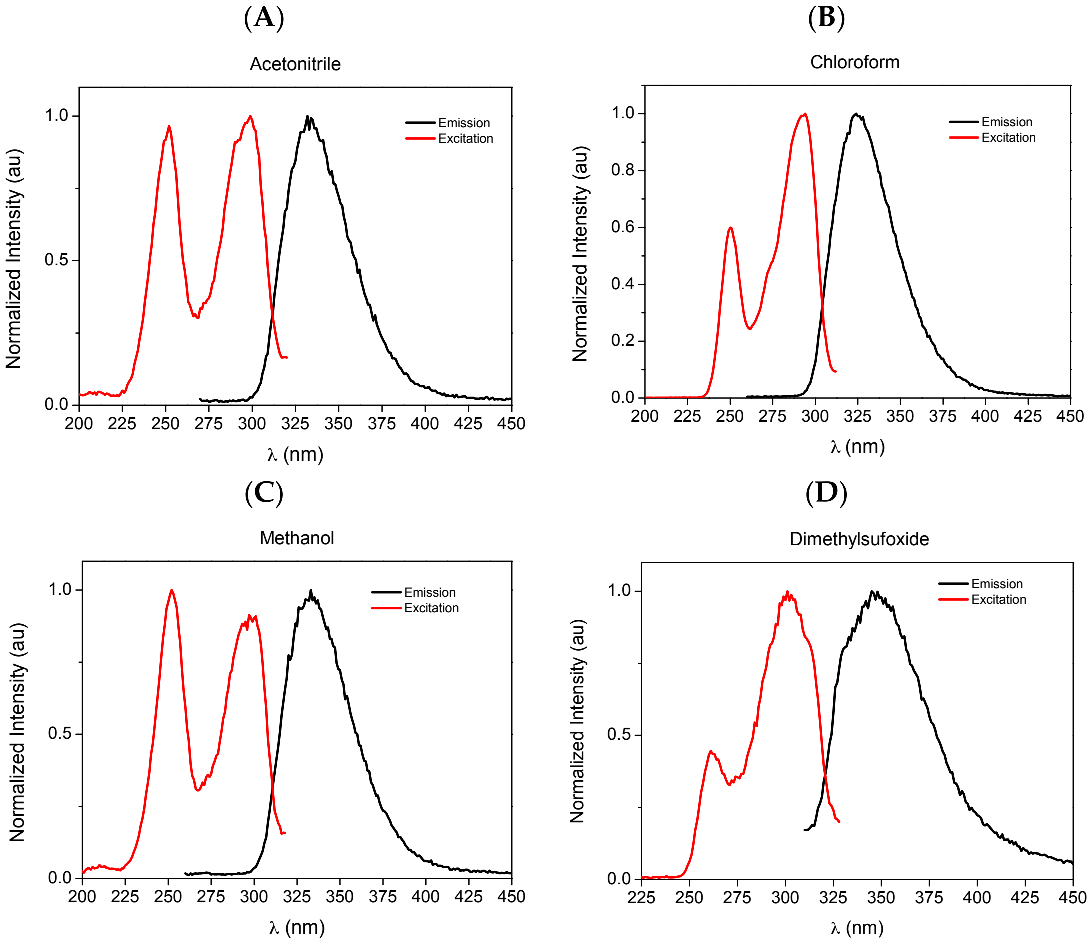

Figure S3. Normalized excitation and emission spectra of **1** (0.01 mM) in (**A**) chloroform, (**B**) acetonitrile, (**C**) methanol and (**D**) dimethylsulfoxide at room temperature under aerobic conditions.

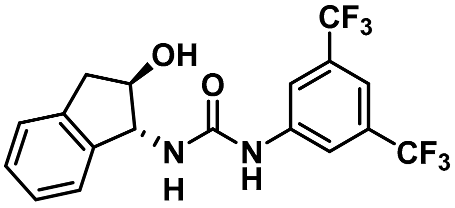

2

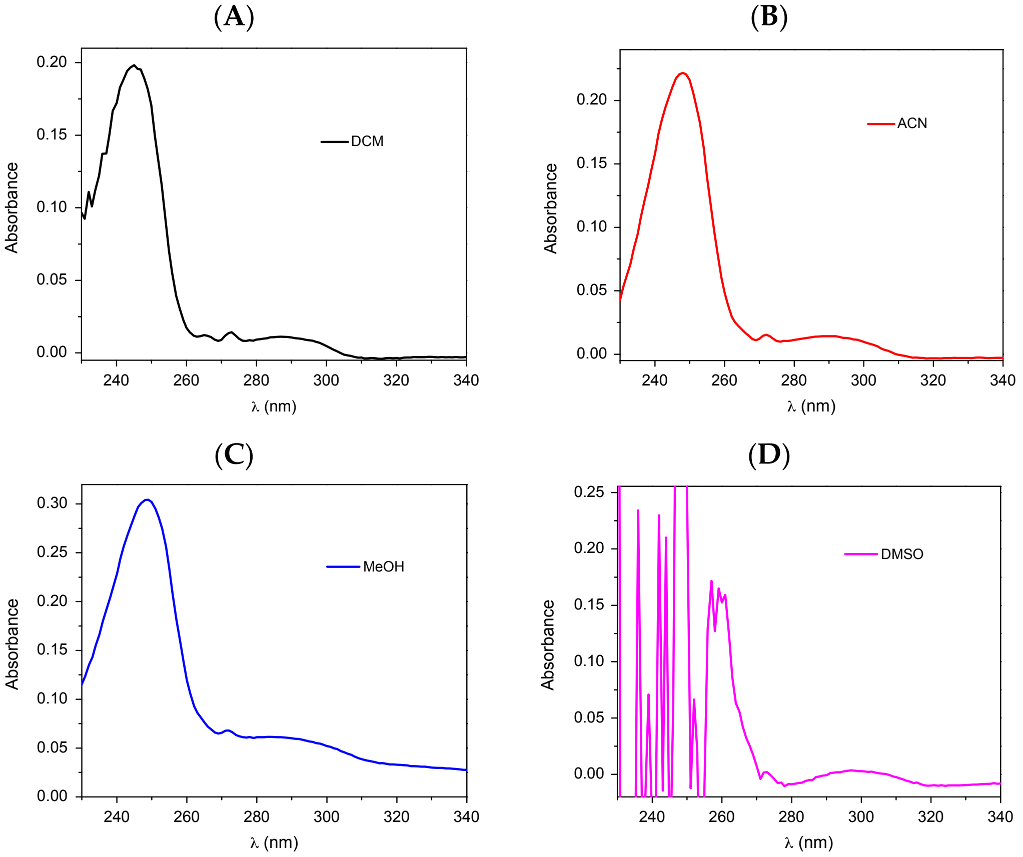

Figure S4. Absorption spectra of **2** (0.01 mM) in (**A**) dichloromethane, (**B**) acetonitrile, (**C**) methanol and (**D**) dimethylsulfoxide at room temperature under aerobic conditions.

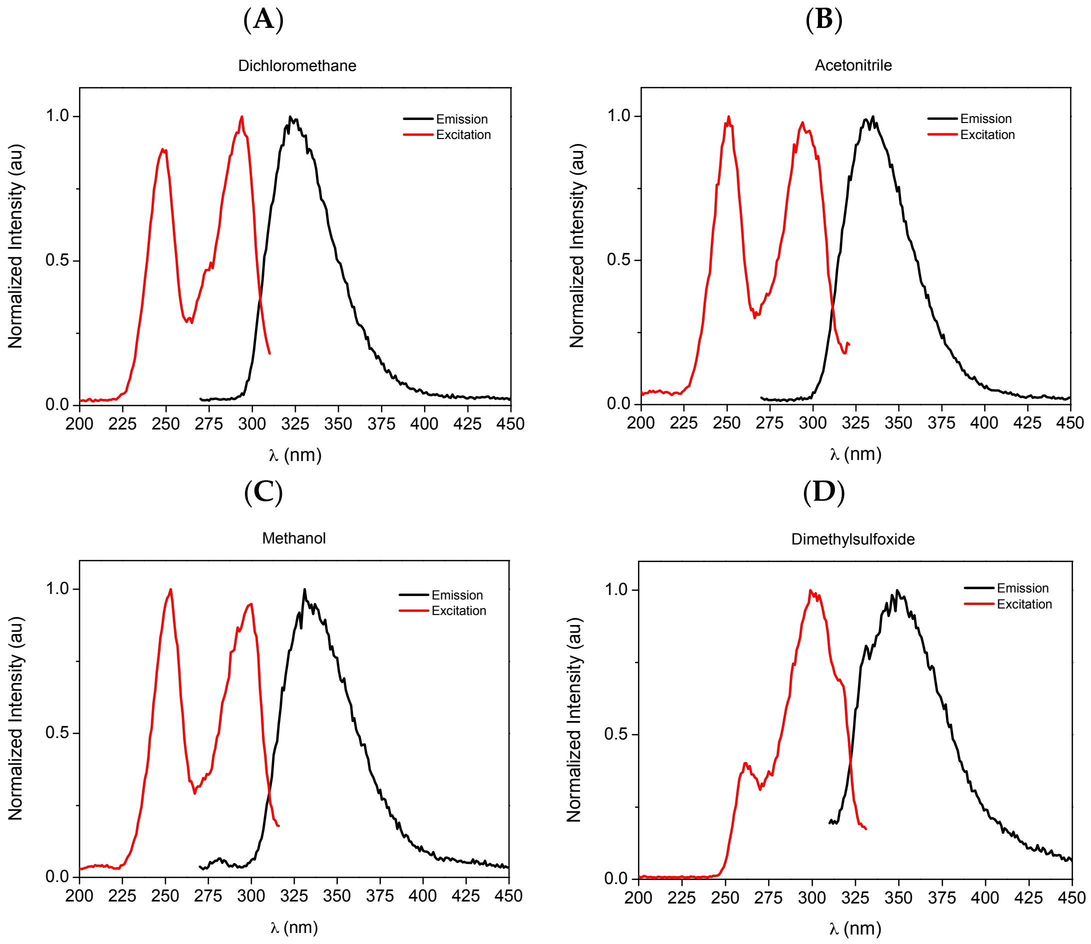

Figure S5. Normalized excitation and emission spectra of **2** (0.01 mM) in (**A**) dichloromethane, (**B**) acetonitrile, (**C**) methanol and (**D**) dimethylsulfoxide at room temperature under aerobic conditions.

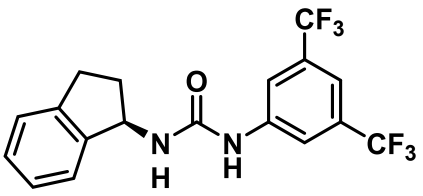

3

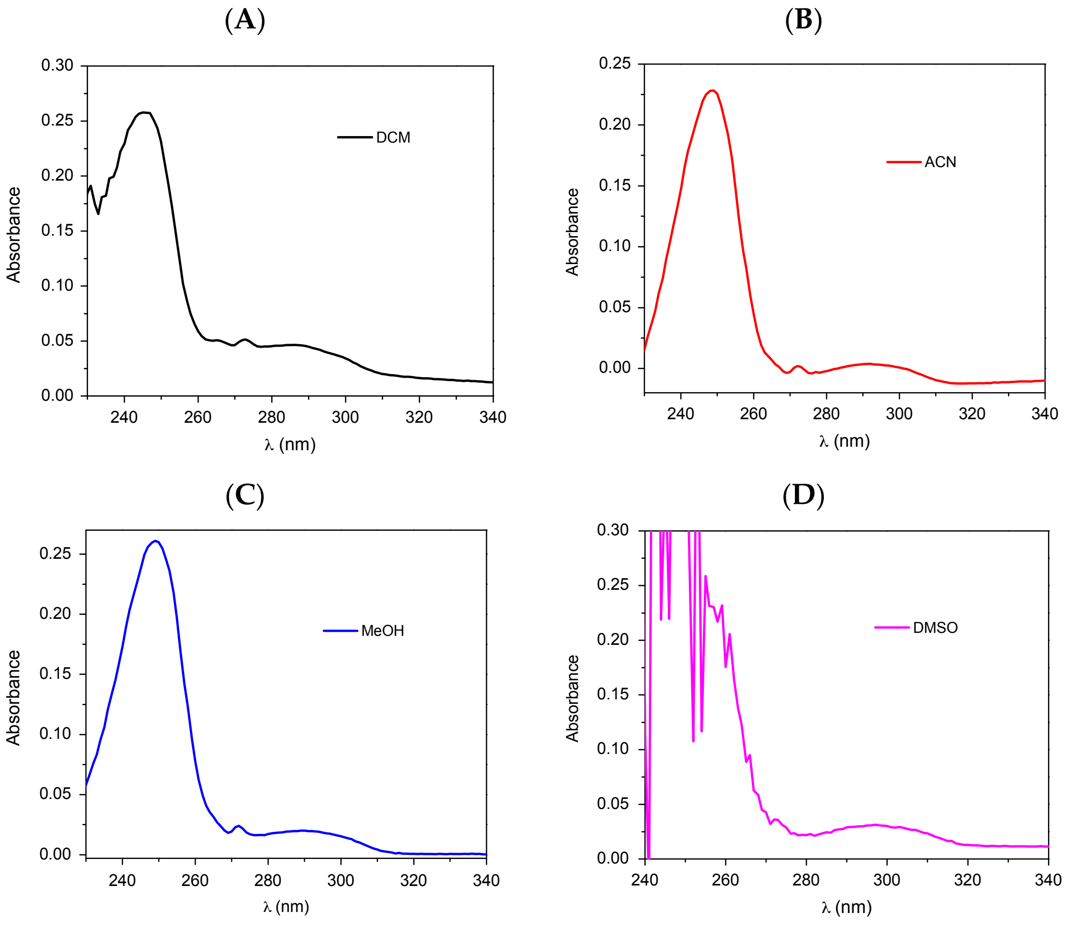

Figure S6. Absorption spectra of **3** (0.01 mM) in (**A**) dichloromethane, (**B**) acetonitrile, (**C**) methanol and (**D**) dimethylsulfoxide at room temperature under aerobic conditions.

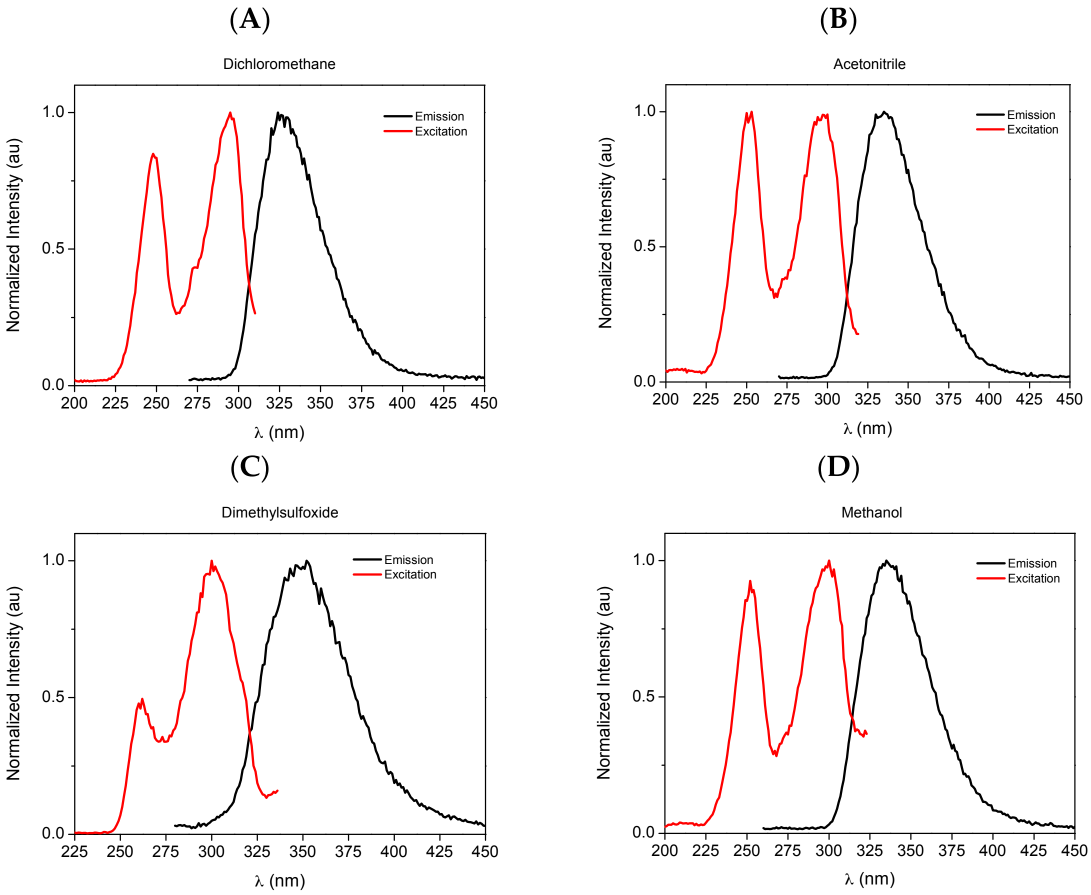

Figure S7. Normalized excitation and emission spectra of **3** (0.01 mM) in (**A**) dichloromethane, (**B**) acetonitrile, (**C**) methanol and (**D**) dimethylsulfoxide at room temperature under aerobic conditions.

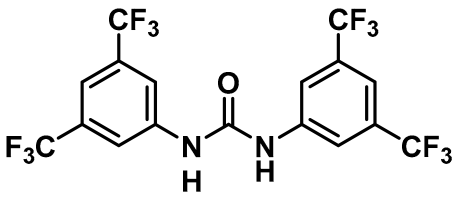

4

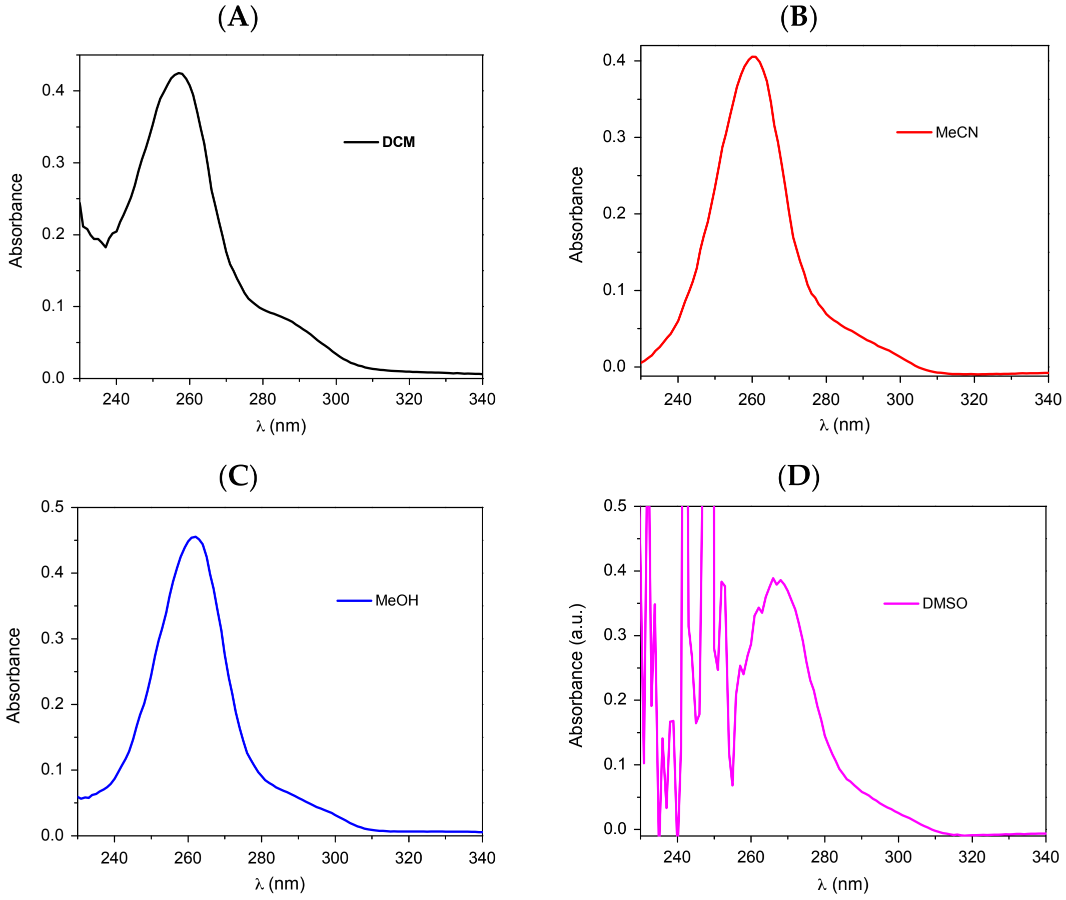

Figure S8. Absorption spectra of **4** (0.01 mM) in (**A**) dichloromethane, (**B**) acetonitrile, (**C**) methanol and (**D**) dimethylsulfoxide at room temperature under aerobic conditions.

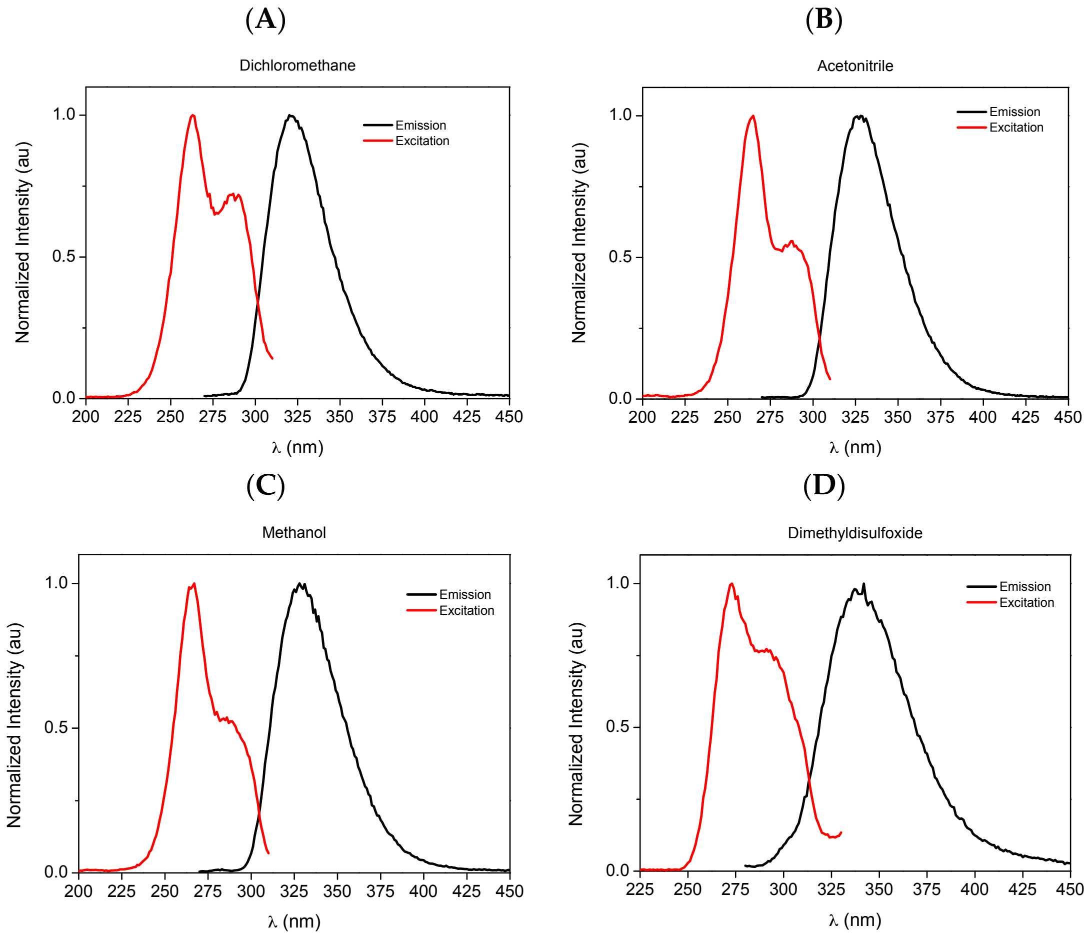

Figure S9. Normalized excitation and emission spectra of **4** (0.01 mM) in (**A**) dichloromethane, (**B**) acetonitrile, (**C**) methanol and (**D**) dimethylsulfoxide at room temperature under aerobic conditions.

Table S1. Photophysical data of sensor **2** ($c = 10^{-5}$ M).

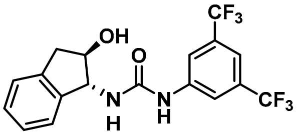

2

**Solvent**	**λ_{abs} (nm) ($\pi\pi^*$/$n\pi^*$)**	**log ε ($M^{-1}\cdot cm^{-1}$) ($\pi\pi^*$)**	**λ_{em} (nm)**	**Stokes (cm^{-1})**	**Singlet Energy (eV)**
DCM	248/294	4.28	322	2957	4.07
ACN	251/294	4.38	332	4162	3.98
MeOH	253/300	4.42	335	3121	3.99
DMSO	-/299	-	349	4791	3.95

Table S2. Photophysical data of sensor **3** ($c = 10^{-5}$ M).

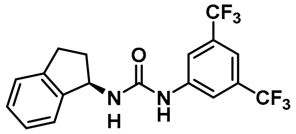

3

**Solvent**	**λ_{abs} (nm) ($\pi\pi^*$/$n\pi^*$)**	**log ε ($M^{-1}\cdot cm^{-1}$) ($\pi\pi^*$)**	**λ_{em} (nm)**	**Stokes (cm^{-1})**	**Singlet Energy (eV)**
DCM	248/295	4.40	324	3034	4.05
ACN	253/297	4.28	335	3819	3.97
MeOH	253/300	4.39	335	3482	3.94
DMSO	-/300	-	350	4761	3.86

Table S3. Photophysical data of sensor **4** ($c = 10^{-5}$ M).

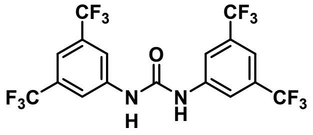

4

**Solvent**	**λ_{abs} (nm) ($\pi\pi^*$/$n\pi^*$)**	**log ε ($M^{-1}\cdot cm^{-1}$) ($\pi\pi^*$)**	**λ_{em} (nm)**	**Stokes (cm^{-1})**	**Singlet Energy (eV)**
DCM	263/287	4.54	320	3593	4.11
ACN	265/288	4.54	328	4234	4.08
MeOH	267/286	4.58	328	4477	4.07
DMSO	-/291	-	340	4952	3.96

(**A**)

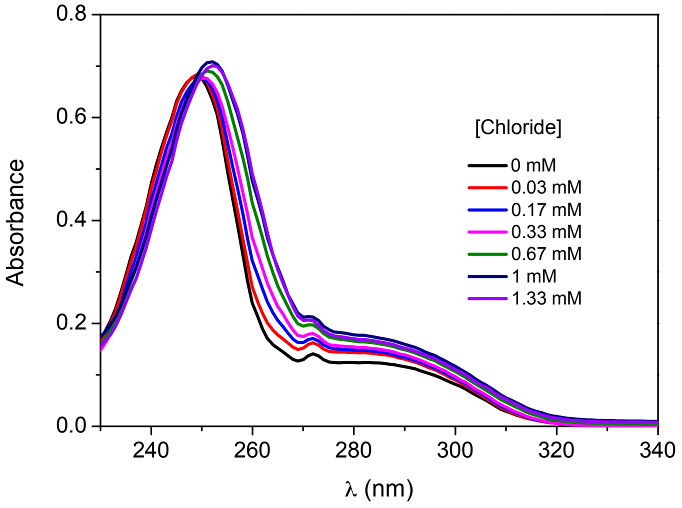

(**B**)

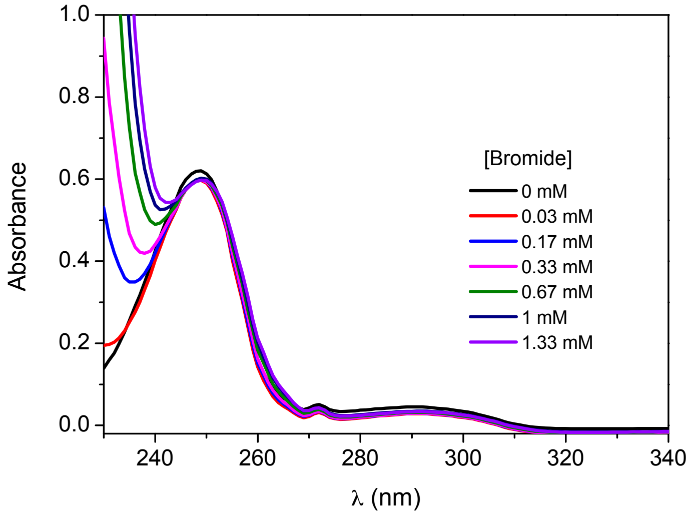

(**C**)

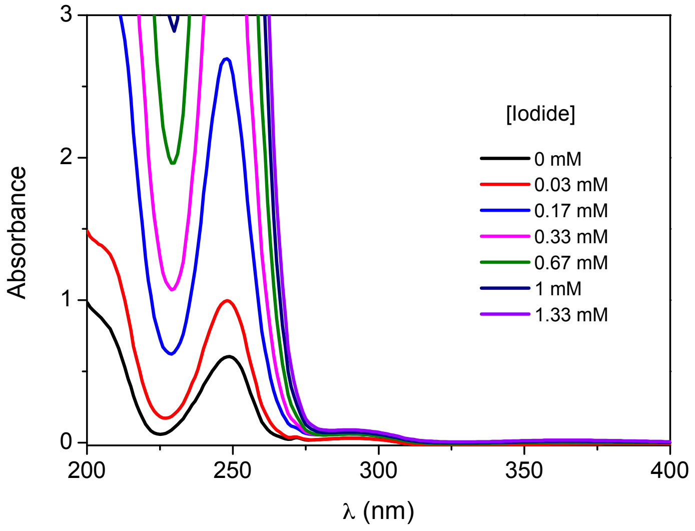

Figure S10. Absorption spectra of **1** (c = 0.04 mM) in the absence and with increasing amounts of (**A**) Cl^-, (**B**) Br^- and (**C**) I^- (c = 0, 0.03 mM → 1 mM) in acetonitrile at room temperature.

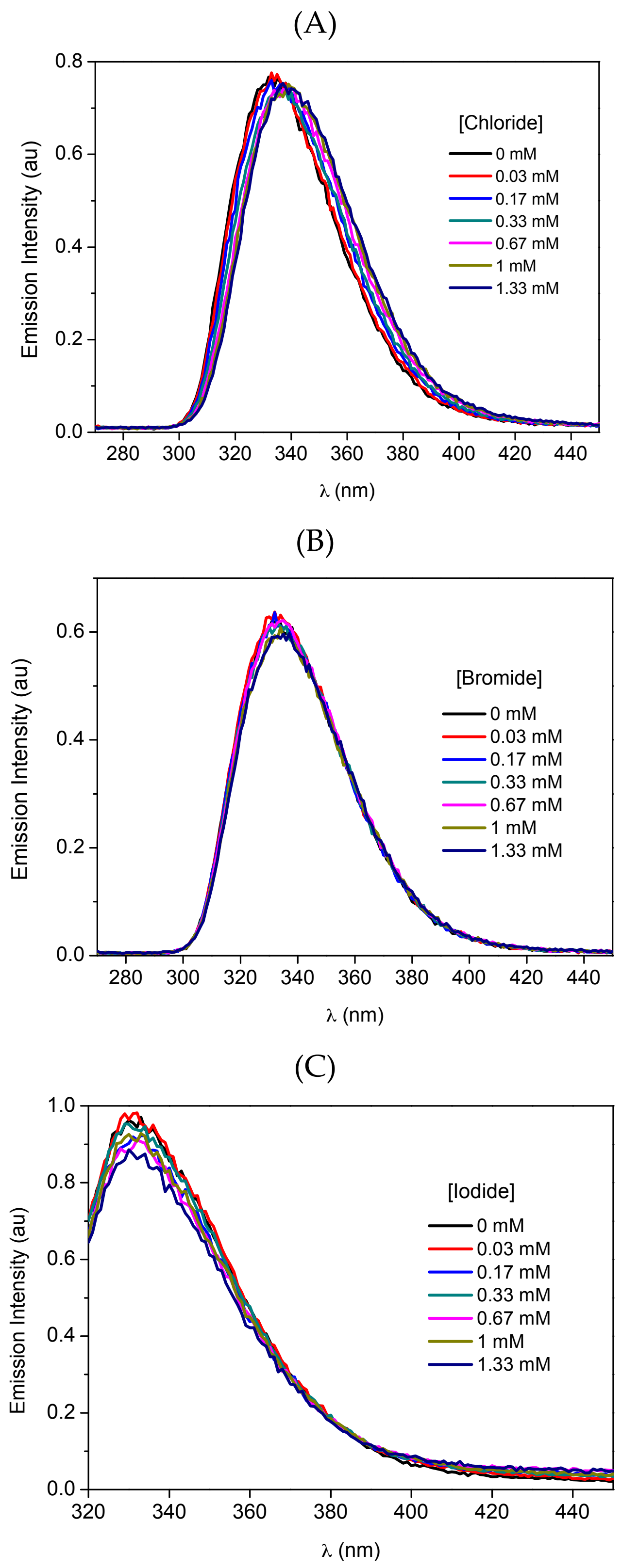

Figure S11. Emission spectra of **1** (c = 0.04 mM) in the presence of increasing amounts of (**A**) chloride (λ_{exc} = 252 nm), (**B**) bromide (λ_{exc} = 252 nm) and (**C**) iodide (λ_{exc} = 300 nm) in acetonitrile.

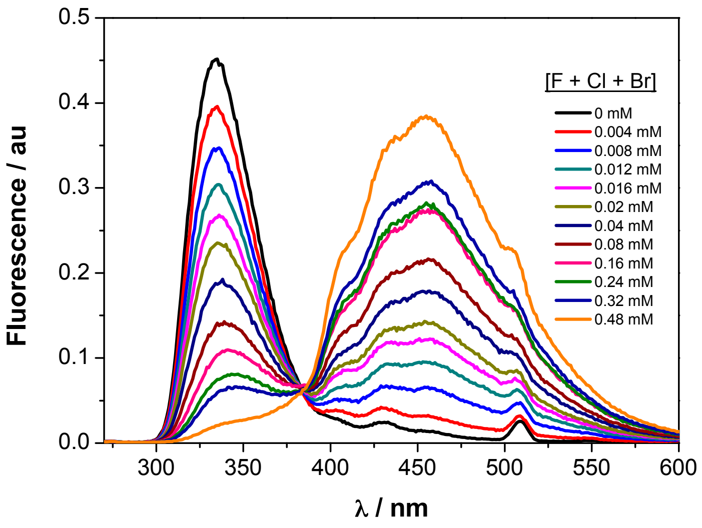

Figure S12. Emission spectra of **1** (c = 0.04 mM, λ_{exc} = 252 nm) in the presence of increasing amounts of a mixture of halide anions (c = 0, 0.004 mM → 0.48 mM) in acetonitrile.

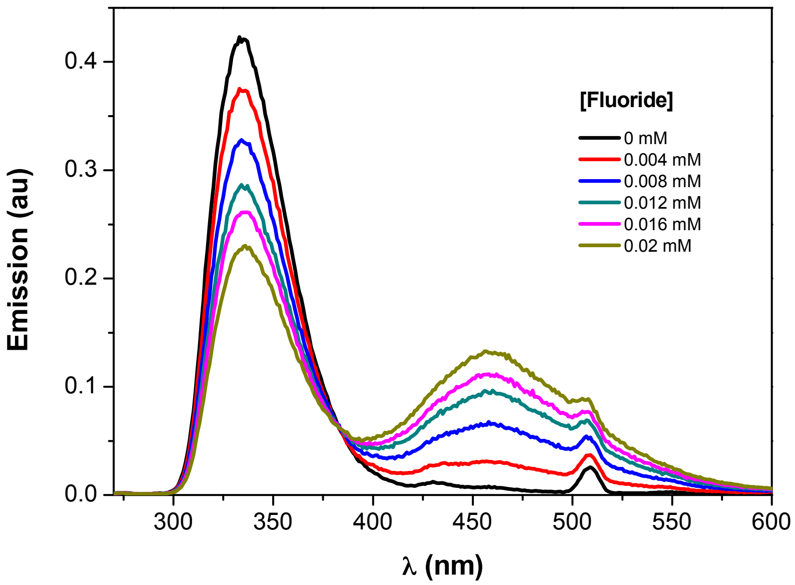

Figure S13. Emission spectra of **1** (c = 0.04 mM, λ_{exc} = 252 nm) in the presence of increasing amounts of fluoride (c = 0, 0.004 mM → 0.02 mM) in acetonitrile.

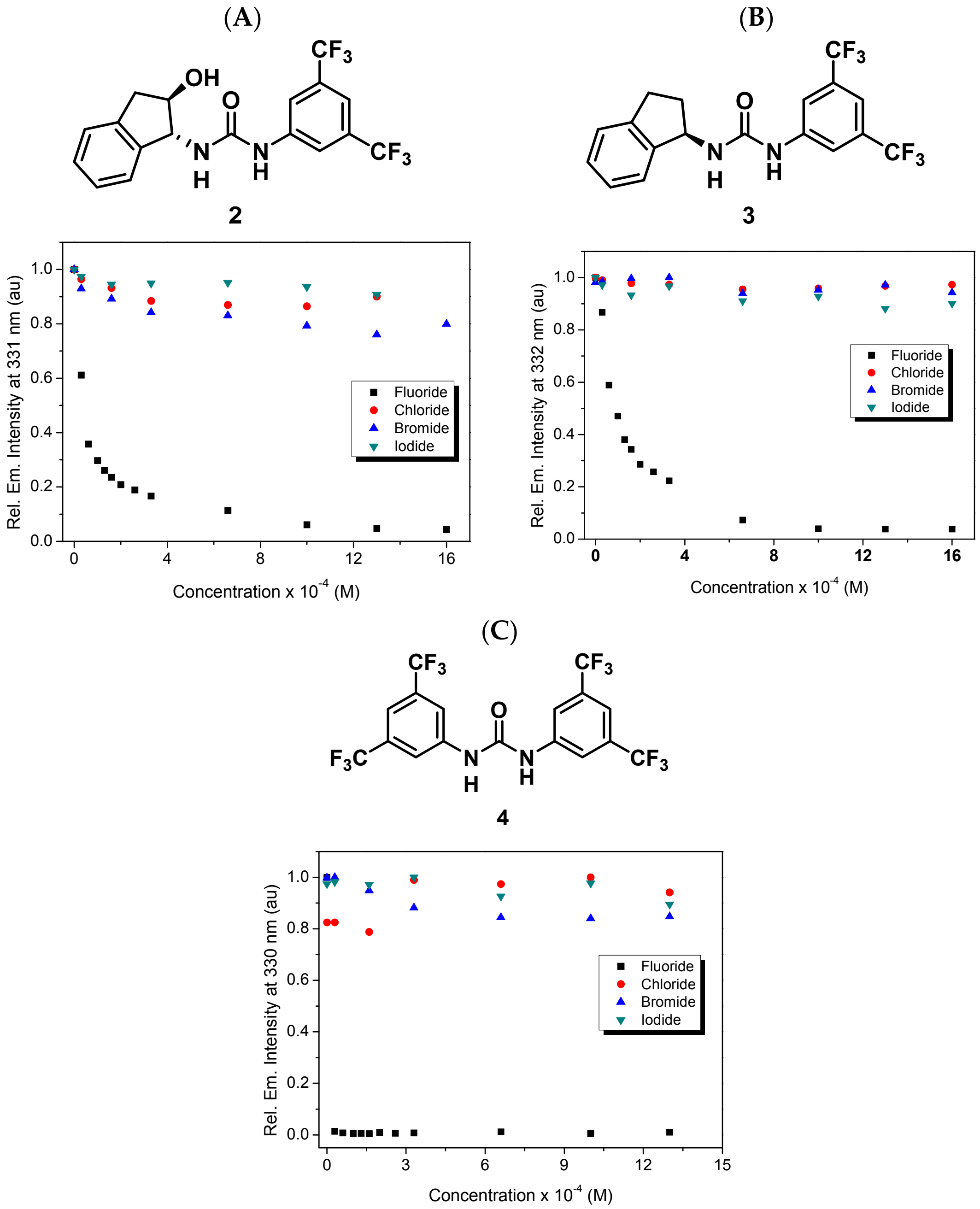

Figure S14. Changes in the maximum emission of sensors (**A**) **2**, (**B**) **3**, (**C**) **4** upon titration with different halides in acetonitrile at room temperature.

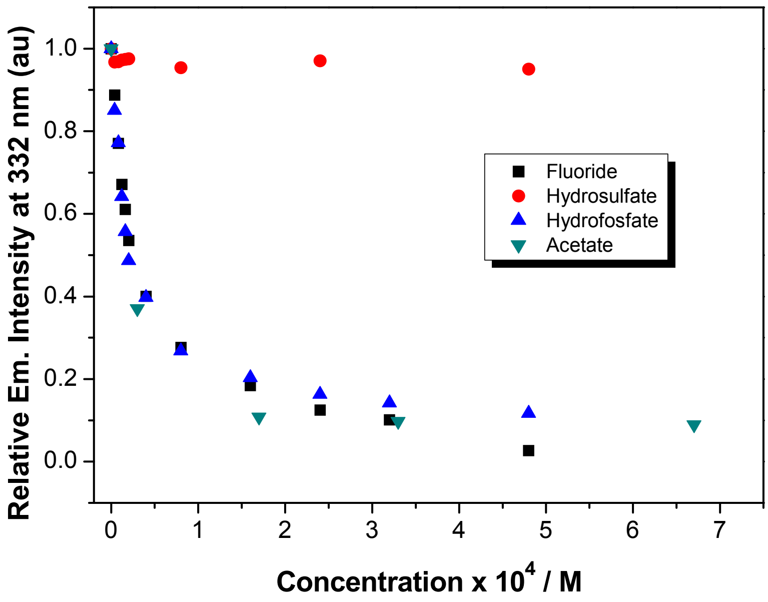

Figure S15. Changes in the emission of **1** (c = 0.04 mM, λ_{exc} = 252 nm) at 332 nm upon titration with different anions in acetonitrile.

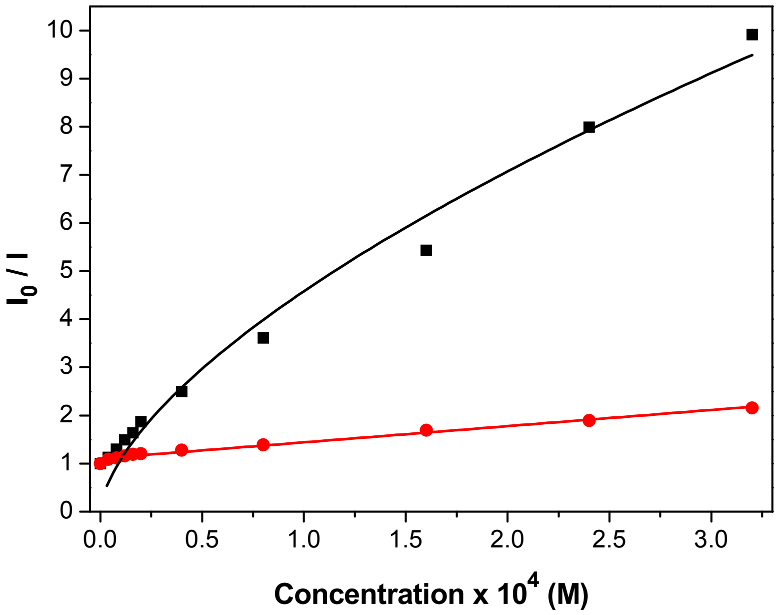

Figure S16. Stern-Volmer plots for the fluorescence of **1** (c = 0.04 mM, λ_{exc} = 252 nm) upon fluoride (■) and DBU (●) titration (c = 0, 0.004 mM → 0.32 mM) in acetonitrile.

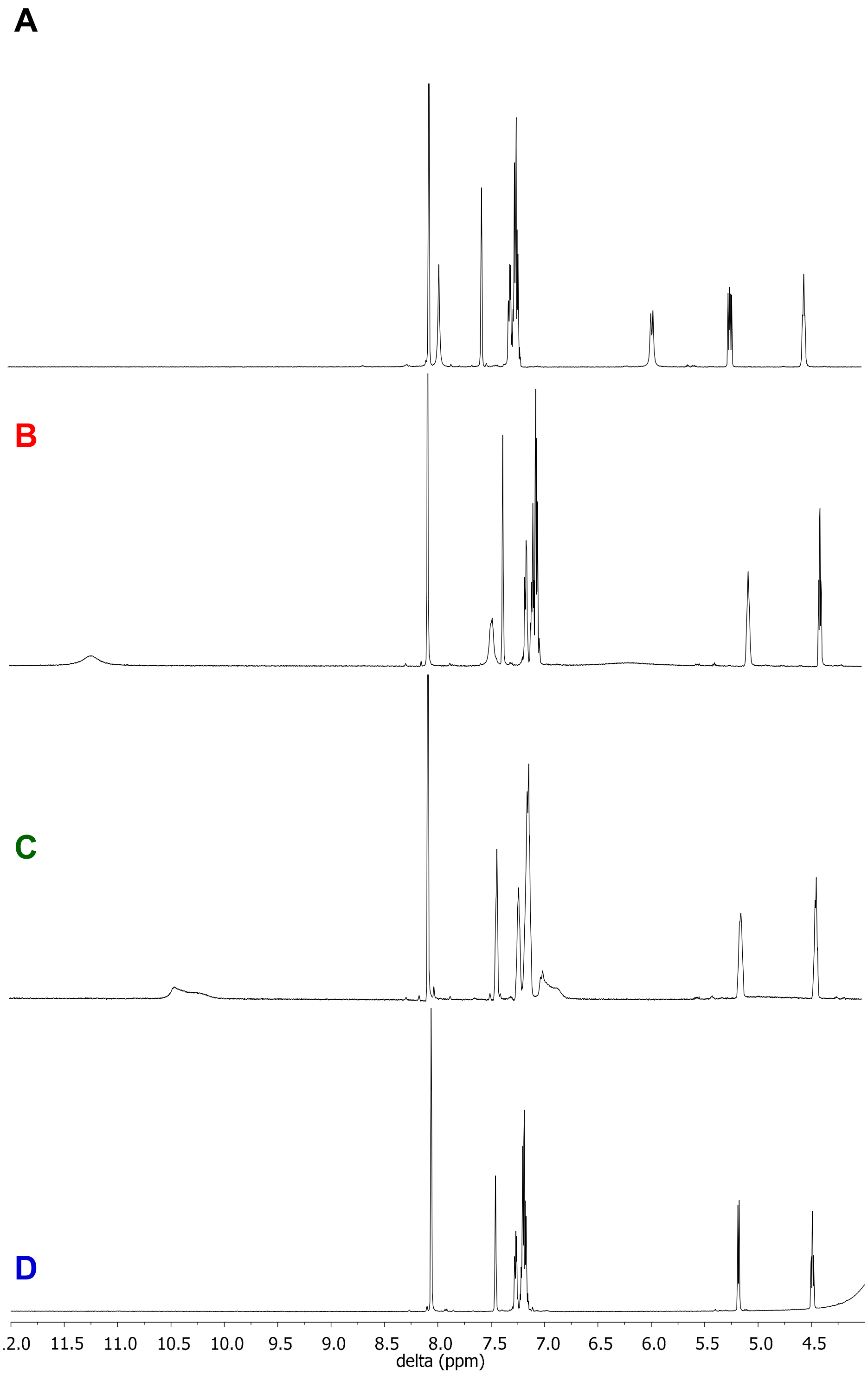

Figure S17. Changes in the ^{1}H-NMR (400 MHz) spectra in CD_3CN. (**A**) Sensor **1** (0.02 mmol), (**B**) sensor **1** + 0.75 eq. of fluoride, (**C**) sensor **1** + 0.75 eq. of fluoride + one drop of MeOH, (D) sensor **1** + one drop of DBU.

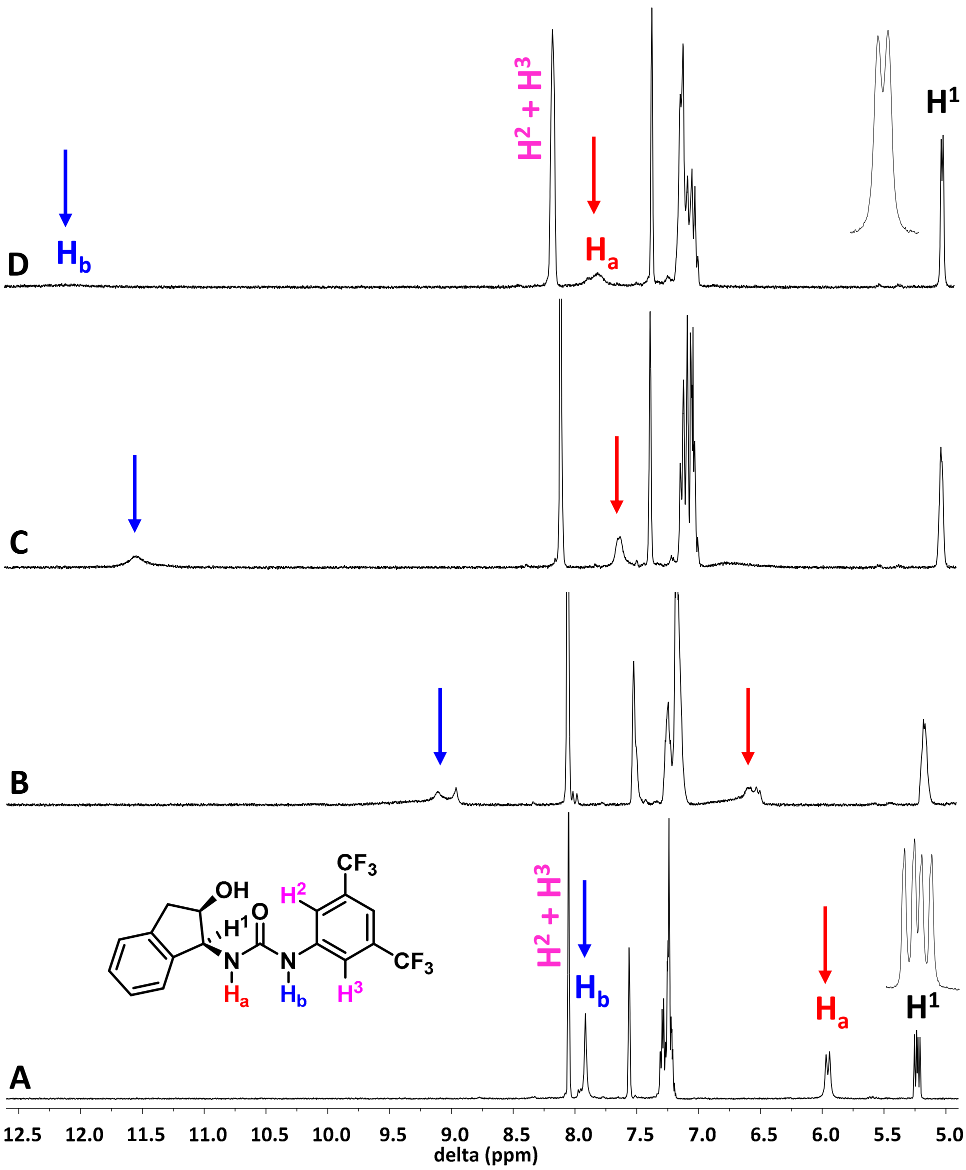

Figure S18. Changes in the ^{1}H NMR (300 MHz) spectra of **1** in CD_3CN upon addition of F^- (**A**) 0 eq, (**B**) 0.25 eq, (**C**) 0.75 eq, (**D**) 1 eq) at room temperature.

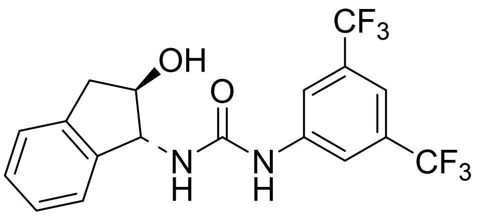

2

(**A**) (**B**)

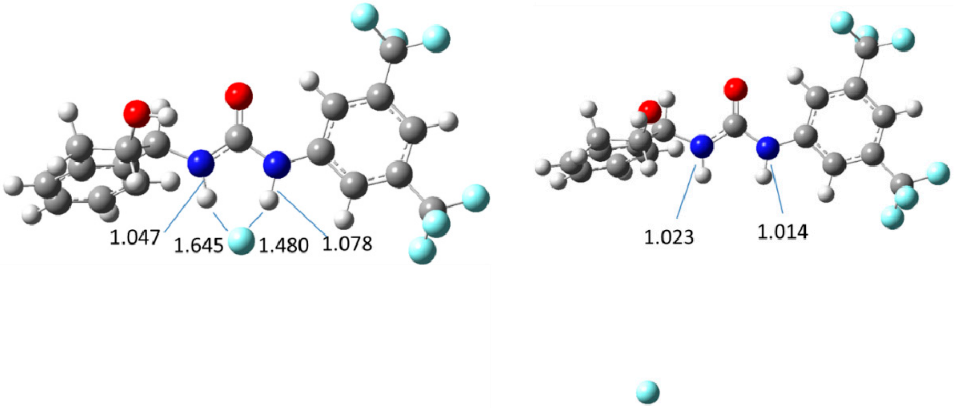

Figure S19. Geometries of sensor **2** fluoride bonded (**A**) and non-bonded fluoride (**B**).

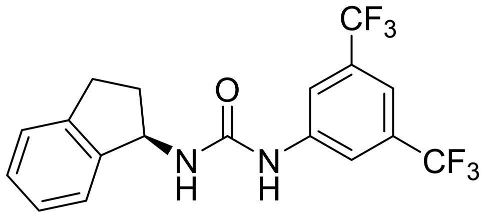

3

(**A**) (**B**)

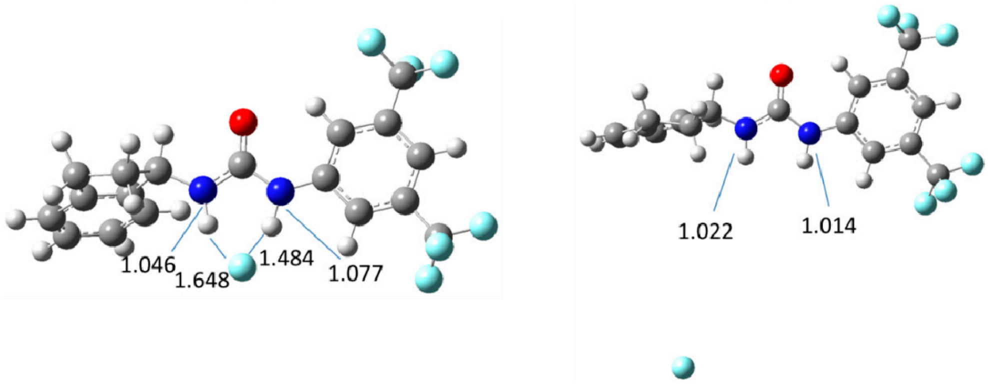

Figure S20. Geometries of sensor **3** fluoride bonded (**A**) and non-bonded fluoride (**B**).

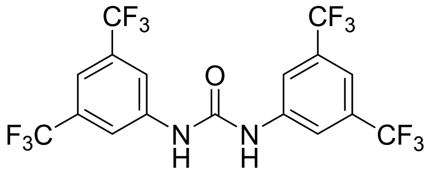

4

(**A**) (**B**)

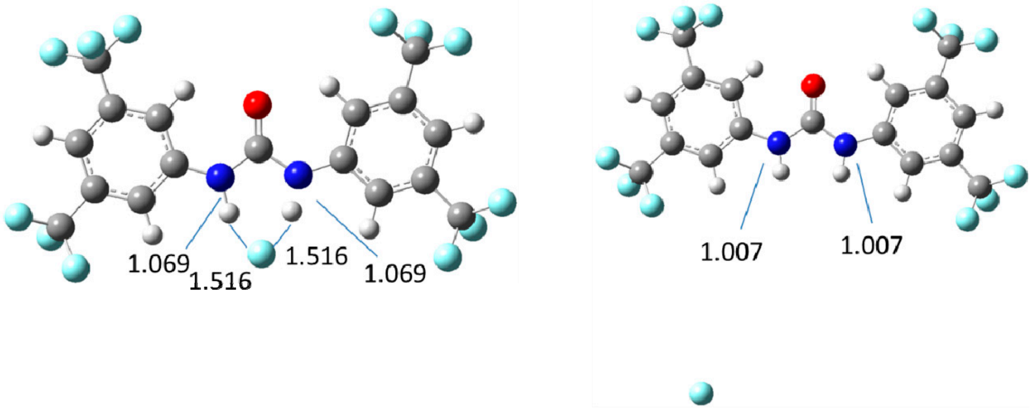

Figure S21 Geometries of sensor **4** fluoride bonded (**A**) and non-bonded fluoride (**B**).

[**1**–F]

6	6.443693	0.703376	0.887840
6	5.842143	0.104523	−0.220898
6	4.440910	0.037735	−0.319021
6	3.627100	0.558385	0.686089
6	4.230579	1.157316	1.804610
6	5.628190	1.231233	1.903099
6	6.483649	−0.535622	−1.439819
6	5.331775	−1.379590	−2.045230
6	4.040388	−0.613485	−1.640614
7	2.839569	−1.418483	−1.626610
6	1.712174	−1.032406	−2.298634
8	1.622516	0.041647	−2.975932
8	5.262713	−2.691932	−1.405757
7	0.684485	−1.957702	−2.150676
6	−0.577059	−1.913501	−2.715879
6	−1.453501	−2.982449	−2.422207
6	−2.741488	−3.014702	−2.957484
6	−3.207114	−1.998532	−3.797487
6	−2.336519	−0.942232	−4.086828
6	−1.042715	−0.881716	−3.563725
6	−2.833063	0.192878	−4.913328
9	−3.793249	−0.198426	−5.847429
6	−3.606714	−4.186650	−2.649199
9	−3.650357	−4.475894	−1.281805
9	−1.820437	0.834622	−5.623835
9	−3.445731	1.193997	−4.144340
9	−4.924472	−4.017357	−3.063154
9	−3.155635	−5.365773	−3.258149
9	1.806073	−3.625930	−0.656107
1	2.780937	−2.335435	−1.133508

1	0.963855	−2.782125	−1.510534
1	−1.107635	−3.777658	−1.770851
1	−0.375961	−0.064187	−3.795357
1	−4.204079	−2.032915	−4.213506
1	5.413157	−1.496968	−3.130914
1	2.545891	0.494600	0.609719
1	7.355004	−1.155825	−1.202750
1	7.525408	0.754098	0.973853
1	3.611887	1.562813	2.599281
1	6.083517	1.693200	2.773753
1	3.875214	0.176222	−2.386126
1	5.985750	−3.259036	−1.740167
1	6.813705	0.225553	−2.161490

1+F

6	−1.097960	−0.790257	−3.440108
6	−0.539592	−1.912079	−2.804113
6	−1.323321	−3.058142	−2.611974
6	−2.646783	−3.090331	−3.041229
6	−3.221216	−1.987747	−3.678471
6	−2.428770	−0.847157	−3.867522
7	0.781826	−1.989576	−2.318785
6	1.785532	−1.024383	−2.363896
8	1.633378	0.133786	−2.864836
6	−3.416498	−4.357334	−2.866759
9	−3.129675	−5.309626	−3.855470
6	−3.043860	0.368269	−4.469509
9	−3.553042	1.248205	−3.504876
7	2.949823	−1.449475	−1.787295
6	4.156593	−0.626882	−1.771516
6	4.461589	0.086791	−0.457171
6	5.842400	0.033927	−0.198460
6	6.564841	−0.730280	−1.288890
6	5.425391	−1.496914	−2.005606
6	3.595246	0.748012	0.412101
6	4.127151	1.359932	1.558942
6	5.505530	1.310072	1.820832
6	6.372267	0.644719	0.940711
8	5.165814	−2.764418	−1.347161
9	−4.119759	0.066882	−5.303984
9	−2.141583	1.113663	−5.222613
9	−3.126518	−4.987025	−1.655147
9	−4.791025	−4.163925	−2.905040
9	4.628435	−8.162443	4.394839
1	3.088493	−2.393135	−1.433991
1	1.015456	−2.867283	−1.877634
1	−0.905642	−3.919561	−2.120714
1	−0.492679	0.091292	−3.588568
1	−4.249298	−2.013734	−4.013063
1	5.619552	−1.665732	−3.070071
1	2.526426	0.790982	0.218504
1	7.328574	−1.420010	−0.913361
1	7.437696	0.601733	1.147895
1	3.466660	1.874119	2.249992

1	5.902167	1.785110	2.712716
1	4.060297	0.110506	−2.576962
1	5.756356	−3.463620	−1.681919
1	7.059645	−0.042291	−1.987932

1+F

6	−1.097960	−0.790257	−3.440108
6	−0.539592	−1.912079	−2.804113
6	−1.323321	−3.058142	−2.611974
6	−2.646783	−3.090331	−3.041229
6	−3.221216	−1.987747	−3.678471
6	−2.428770	−0.847157	−3.867522
7	0.781826	−1.989576	−2.318785
6	1.785532	−1.024383	−2.363896
8	1.633378	0.133786	−2.864836
6	−3.416498	−4.357334	−2.866759
9	−3.129675	−5.309626	−3.855470
6	−3.043860	0.368269	−4.469509
9	−3.553042	1.248205	−3.504876
7	2.949823	−1.449475	−1.787295
6	4.156593	−0.626882	−1.771516
6	4.461589	0.086791	−0.457171
6	5.842400	0.033927	−0.198460
6	6.564841	−0.730280	−1.288890
6	5.425391	−1.496914	−2.005606
6	3.595246	0.748012	0.412101
6	4.127151	1.359932	1.558942
6	5.505530	1.310072	1.820832
6	6.372267	0.644719	0.940711
8	5.165814	−2.764418	−1.347161
9	−4.119759	0.066882	−5.303984
9	−2.141583	1.113663	−5.222613
9	−3.126518	−4.987025	−1.655147
9	−4.791025	−4.163925	−2.905040
9	4.628435	−8.162443	4.394839
1	3.088493	−2.393135	−1.433991
1	1.015456	−2.867283	−1.877634
1	−0.905642	−3.919561	−2.120714
1	−0.492679	0.091292	−3.588568
1	−4.249298	−2.013734	−4.013063
1	5.619552	−1.665732	−3.070071
1	2.526426	0.790982	0.218504
1	7.328574	−1.420010	−0.913361
1	7.437696	0.601733	1.147895
1	3.466660	1.874119	2.249992
1	5.902167	1.785110	2.712716
1	4.060297	0.110506	−2.576962
1	5.756356	−3.463620	−1.681919
1	7.059645	−0.042291	−1.987932

2+F

6	6.520822	0.461383	1.006385
6	5.881556	0.027823	−0.156908
6	4.480237	0.101881	−0.265503
6	3.705202	0.609920	0.776916

6	4.348008	1.046055	1.946569
6	5.744504	0.971383	2.059685
6	6.473636	−0.536604	−1.432672
6	5.263693	−1.242216	−2.097330
6	4.028763	−0.423429	−1.622157
7	2.794899	−1.196578	−1.581053
6	1.690415	−0.951968	−2.345614
8	1.590158	0.036438	−3.122295
1	5.170113	−2.278505	−1.796653
7	0.682822	−1.901822	−2.172784
6	−0.593463	−1.913148	−2.751115
6	−1.048966	−0.932105	−3.651846
6	−2.341746	−1.025765	−4.175857
6	−3.206900	−2.067014	−3.830880
6	−2.742927	−3.034568	−2.935936
6	−1.456043	−2.968004	−2.399764
6	−3.606431	−4.196147	−2.580128
9	−4.950219	−3.977000	−2.864439
6	−2.827450	0.063177	−5.070835
9	−1.821610	0.576547	−5.888045
9	−3.249412	−5.363110	−3.267296
9	−3.525673	−4.523694	−1.225630
9	−3.329878	1.160020	−4.358141
9	−3.859713	−0.349976	−5.909561
9	5.277122	−7.827797	3.824020
1	2.819192	−2.007412	−0.957179
1	0.888312	−2.678935	−1.555303
1	−1.122232	−3.726603	−1.700496
1	−0.388324	−0.125534	−3.931705
1	−4.203954	−2.125378	−4.244625
8	5.297434	−1.195136	−3.556178
1	2.623946	0.658120	0.691896
1	7.340946	−1.185845	−1.286829
1	7.600756	0.400074	1.101550
1	3.760518	1.438656	2.770385
1	6.229000	1.305833	2.971650
1	3.868501	0.393853	−2.334654
1	6.791015	0.275717	−2.102829
1	5.982517	−1.807277	−3.892604

[**3**–F]

6	−1.063586	−0.880767	−3.622510
6	−0.596219	−1.856015	−2.736582
6	−1.420520	−2.935779	−2.372678
6	−2.714416	−3.050803	−2.883810
6	−3.186457	−2.069497	−3.769975
6	−2.365322	−0.992299	−4.137621
6	0.755403	−1.960788	−2.050805
6	0.497318	−2.990331	−0.915526
6	−0.686567	−3.876191	−1.419997
7	−1.509776	−4.422443	−0.355394
6	−1.632951	−5.765029	−0.132166
8	−1.048648	−6.660436	−0.821197
1	1.380970	−3.585156	−0.665899

7	−2.480367	−6.022609	0.944860
6	−2.847094	−7.253723	1.457895
6	−2.393318	−8.498083	0.962990
6	−2.827877	−9.684402	1.559574
6	−3.712152	−9.691946	2.642993
6	−4.159565	−8.458302	3.126870
6	−3.740304	−7.257448	2.554148
6	−5.053081	−8.406791	4.316851
9	−5.682779	−9.620313	4.578883
6	−2.397558	−10.982374	0.969298
9	−1.123492	−10.928250	0.405851
9	−4.368833	−8.066856	5.492520
9	−6.062400	−7.449396	4.189458
9	−3.245275	−11.415162	−0.061537
9	−2.381204	−12.021383	1.899820
9	−3.075163	−3.662293	1.585456
1	0.173032	−2.468762	−0.006184
1	−2.057076	−3.817800	0.299254
1	−2.865876	−5.117676	1.383671
1	−4.103072	−6.309991	2.938791
1	−1.708673	−8.503714	0.127800
1	−4.038698	−10.618772	3.093310
1	−3.350923	−3.879556	−2.588900
1	1.119197	−0.998772	−1.673388
1	−0.435292	−0.040108	−3.903370
1	−4.193348	−2.141262	−4.169706
1	−2.741730	−0.235698	−4.819434
1	−0.295508	−4.737269	−1.976877
1	1.515476	−2.335934	−2.751584

3+F

6	6.519924	0.466257	1.010587
6	5.877443	0.042330	−0.167654
6	4.474767	0.130304	−0.248090
6	3.753417	0.634574	0.838004
6	4.381849	1.060937	2.010402
6	5.774371	0.967582	2.078477
7	6.684048	−0.447337	−1.203255
6	6.278872	−0.912032	−2.455946
8	5.070764	−0.994514	−2.809976
6	2.276786	0.788366	0.703098
9	1.621800	0.798994	1.932343
6	6.485257	1.348646	3.331590
9	7.688721	2.010158	3.082479
7	7.320674	−1.274283	−3.258414
6	7.189535	−1.879204	−4.579961
6	7.946356	−1.139995	−5.679172
6	8.518586	−2.059047	−6.577752
6	8.179114	−3.479920	−6.163237
6	7.781894	−3.324818	−4.669560
6	8.100440	0.234689	−5.862829
6	8.846417	0.691881	−6.961221
6	9.420449	−0.221320	−7.858614
6	9.257351	−1.603909	−7.672657

1	7.038174	−4.066978	−4.362054
9	5.727518	2.180036	4.149768
9	6.824875	0.238480	4.115153
9	1.697175	−0.226400	−0.056705
9	1.918139	1.985395	0.068903
9	16.136266	−1.859826	−2.121850
1	8.625652	−3.430908	−3.998126
1	8.282066	−1.188260	−2.921058
1	7.678581	−0.467563	−1.008867
1	7.600205	0.411455	1.084979
1	3.976586	−0.202291	−1.146150
1	3.809837	1.448827	2.841661
1	7.662341	0.939918	−5.162825
1	8.979288	−4.203765	−6.338401
1	9.708194	−2.306168	−8.367856
1	8.983845	1.757687	−7.114242
1	10.000356	0.143579	−8.700726
1	6.115822	−1.900143	−4.799767
1	7.312308	−3.839736	−6.737887

[**4**–F]

6	−5.549110	−0.030841	0.686435
6	−4.937613	−0.024315	1.957762
6	−5.761820	−0.070043	3.103692
6	−7.148625	−0.119448	2.976263
6	−7.763265	−0.131078	1.718772
6	−6.942647	−0.088697	0.588804
7	−3.565504	0.013877	2.169646
6	−2.564384	0.053078	1.208905
8	−2.763431	0.087387	−0.054754
6	−7.990936	−0.244597	4.198200
9	−9.242862	0.351323	4.050467
6	−7.569624	−0.026816	−0.761490
9	−6.796670	−0.640928	−1.745558
7	−1.316347	0.048276	1.816599
6	−0.075038	0.046687	1.194161
6	0.116840	0.044102	−0.203592
6	1.413878	0.039901	−0.724670
6	2.542445	0.032620	0.098626
6	2.343332	0.040381	1.483589
6	1.061664	0.041830	2.031092
6	3.522221	−0.029559	2.391230
9	3.296288	0.588977	3.619771
6	1.594542	−0.037882	−2.201589
9	2.793849	0.531138	−2.628705
9	3.891111	−1.347557	2.694929
9	4.659160	0.561695	1.842646
9	1.614778	−1.358658	−2.672334
9	0.573772	0.595649	−2.907833
9	−7.767862	1.288608	−1.205149
9	−8.825968	−0.629894	−0.800683
9	−8.248307	−1.579378	4.541059
9	−7.400884	0.326393	5.324082
9	−2.090325	0.002408	4.220610

1	−3.180326	0.001266	3.166402
1	−5.303552	−0.060421	4.085500
1	−4.924927	0.000959	−0.194134
1	−8.839922	−0.173167	1.624763
1	−1.377168	0.029199	2.883676
1	0.926716	0.051504	3.106333
1	3.539313	0.041350	−0.320677
1	−0.747824	0.054586	−0.850148
**4**+F			
6	1.084693	−0.034822	2.018017
6	−0.053114	−0.005181	1.198963
6	0.115873	0.040112	−0.194194
6	1.403658	0.060793	−0.729878
6	2.538191	0.027539	0.076658
6	2.357343	−0.014919	1.456853
7	−1.297902	−0.016433	1.848522
6	−2.564578	−0.012674	1.289452
8	−2.788269	0.001722	0.054568
6	1.565518	0.044179	−2.208858
9	0.537296	0.706570	−2.872059
6	3.546473	−0.113488	2.346696
9	4.674565	0.493754	1.801367
7	−3.568566	−0.028781	2.239386
6	−4.950679	−0.050150	2.007635
6	−5.523311	−0.088484	0.727935
6	−6.911918	−0.115333	0.599095
6	−7.757248	−0.099918	1.704129
6	−7.178277	−0.060369	2.972871
6	−5.795508	−0.036876	3.133181
6	−8.048083	−0.123059	4.178754
9	−7.461272	0.472548	5.292375
6	−7.503938	−0.082211	−0.766476
9	−8.771949	−0.654903	−0.815814
9	−9.279120	0.496051	3.981433
9	−8.341963	−1.437420	4.560128
9	−6.723224	−0.744583	−1.707911
9	−7.658209	1.221708	−1.253276
9	3.334315	0.465218	3.594825
9	3.916085	−1.440151	2.602558
9	2.760045	0.628975	−2.622587
9	1.578512	−1.254543	−2.732223
9	−4.510520	0.164668	11.320423
1	−3.293659	−0.027115	3.208656
1	−5.368075	0.007478	4.128881
1	−4.885820	−0.104558	−0.138687
1	−8.830263	−0.120119	1.581530
1	−1.248204	−0.037031	2.853956
1	0.971358	−0.060658	3.092870
1	3.528827	0.057160	−0.352613
1	−0.751085	0.070461	−0.829869
